# High-efficiency color-tunable ultralong room-temperature phosphorescence from organic–inorganic metal halides *via* synergistic inter/intramolecular interactions†

**DOI:** 10.1039/d4sc01630k

**Published:** 2024-05-23

**Authors:** Lei Zhou, Kailei Li, Yuanyuan Chang, Yuan Yao, Yuqi Peng, Ming Li, Rongxing He

**Affiliations:** a Key Laboratory of Luminescence Analysis and Molecular Sensing (Southwest University), Ministry of Education, School of Chemistry and Chemical Engineering, Southwest University Chongqing 400715 China herx@swu.edu.cn; b Institute of Materials Science and Devices, School of Materials Science and Engineering, Suzhou University of Science and Technology Suzhou 215009 China

## Abstract

Materials exhibiting highly efficient, ultralong and multicolor-tunable room-temperature phosphorescence (RTP) are of practical importance for emerging applications. However, these are still very scarce and remain a formidable challenge. Herein, using precise structure design, several novel organic–inorganic metal-halide hybrids with efficient and ultralong RTP have been developed based on an identical organic cation (A). The original organic salt (ACl) exhibits red RTP properties with low phosphorescence efficiency. However, after embedding metals into the organic salt, the changed crystal structure endows the resultant metal–halide hybrids with excellent RTP properties. In particular, A_2_ZnCl_4_·H_2_O exhibits the highest RTP efficiency of up to 56.56% with a long lifetime of up to 159 ms. It is found that multiple inter/intramolecular interactions and the strong heavy-atom effect of the rigid metal–halide hybrids can suppress molecular motion and promote the ISC process, resulting in highly stable and localized triplet excitons followed by highly efficient RTP. More crucially, multicolor-tunable fluorescence and RTP achieved by tuning the metal and halogen endow these materials with wide application prospects in the fields of multilevel information encryption and dynamic optical data storage. The findings promote the development of phosphorescent metal-halide hybrids for potential high-tech applications.

## Abstract 2

A high-efficiency, color-tunable ultralong RTP system based on metal-halide hybrids has been developed, which shows forward-looking application prospects in the fields of information encryption, anti-counterfeiting and dynamic optical data storage.

## Graphical abstract

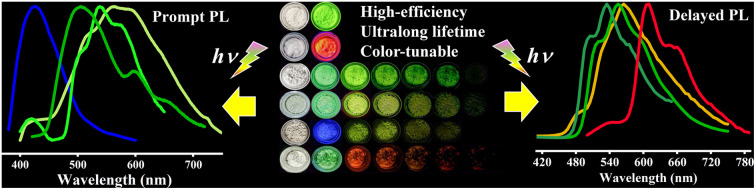

## Introduction

Advanced phosphorescent materials with ultralong phosphorescence (>100 ms) and high efficiency have aroused considerable attention because of their broad applications in advanced anti-counterfeiting,^1,2^ chemical sensors,^3,4^ lighting and display,^5^ bioimaging,^6–8^ and organic electronics^9^ applications, among others. Generally, molecular phosphorescence is observed at very low temperatures due to the forbidden triplet transition, while room-temperature phosphorescence (RTP) is difficult to achieve at ambient conditions due to the presence of oxygen and the thermal-quenching effect. Most RTP systems containing noble metals might suffer some drawbacks, such as high cost, potential toxicity and harsh preparation conditions, rendering them less competitive compared with ultralong organic phosphorescence (UOP) materials. Pure organic RTP phosphors have gained much attention due to their good processability, low cost, high efficiency and tunable energy level.^1,6,10–12^ However, tunable UOP systems usually require complicated molecular designs and tedious chemical syntheses.^13,14^ Thus, advanced phosphorescent materials with easily tunable structures and facile synthesis approaches are highly desired.

Low-dimensional organic–inorganic metal-halide hybrids have shown excellent optoelectronic properties in applications like solar cells and photoemission devices.^15–26^ For example, zero-dimensional (0D) metal halides assembled from inorganic metal-halide units (anion group) and organic cations at the molecular level can promote excited-state carriers localized within the inorganic units, thereby resulting in effective radiative recombination.^27^ Generally, versatile structures and intermolecular interactions can be achieved for 0D metal-halide hybrids through selection of the metal, halogen and organic components, which provides suitable platforms to design materials with tunable optical properties. To date, however, ultralong RTP for 0D metal-halide hybrids has attracted less attention.^28–31^ For 0D metal-halide hybrids, the metal-halide unit can coordinate strongly with the surrounding organic cations to form a rigid structure, and moreover, abundant halide ions can lead to multiple noncovalent interactions by forming halogen bonds, hydrogen bonds, *etc.* Therefore, the anticipated severe restriction of the rotation and vibration of organic cations and the modulation of spin–orbital coupling (SOC) can be realized in 0D metal-halide hybrids, which are conductive to highly efficient RTP output.

Up to now, RTP materials with high phosphorescence quantum yields (QY_phos._) and ultralong lifetime (*τ*) are still rare, due to the inherent competition between QY_phos._ and *τ*.^29^ This challenge limits the potential for long-afterglow emission applications. It is a fact that the RTP properties are highly dependent on inter/intramolecular interactions. For example, strong intermolecular halogen and hydrogen bonding in a system can accelerate the inter-system crossing (ISC) process and lead to enhanced RTP efficiency.^32,33^ Moreover, a strong heavy-atom effect can also greatly increase SOC and promote the ISC process.^34,35^ As such, various design strategies have been proposed to improve RTP properties through inter/intramolecular interactions, including crystal engineering,^29,36^ H-aggregation,^1,37^ polymerization,^38^ host–guest doping,^15,39^ electronic coupling,^40,41^ metal–organic frameworks (MOFs),^42,43^ and supramolecular self-assemblies.^44–46^ 0D metal-halide hybrids can provide rigid structure, heavy-atom effect and multiple intermolecular interactions through manipulation of the center metals and halogen (Cl, Br and I).^43,47,48^ Thus, materials with tunable RTP properties, high QY_phos._ (>20%) and long lifetime (*τ* > 100 ms) are expected to be realized; however, such materials are still rarely developed due to the inherent competition among these properties.

To validate our hypothesis, a series of 0D metal-halide hybrids were used as a model system to meet the challenge of developing tunable RTP materials with both high-efficiency and long lifetime. Compared with the organic chloride salt (2-aminoacetophenone chloride, abbreviated as “ACl”), which exhibits green fluorescence and red RTP characteristics, the resultant 0D metal-halide hybrids (A_2_H_3_OInCl_6_·H_2_O, A_2_SnCl_6_ and A_2_ZnCl_4_·H_2_O) prepared by embedding metals into ACl show greatly enhanced RTP efficiency and tunable emission color from blue to red in the visible light range. Specifically, A_2_ZnCl_4_·H_2_O exhibits a long lifetime of 159 ms, the highest ISC rate (*K*_isc_: 3.4 × 10^8^ s^−1^) and the highest QY_phos._ of up to 56.56%. Experimental and computational studies reveal that the embedding of metal to construct 0D metal-halide hybrids can consolidate the structure, induce the heavy-atom effect and trigger multiple noncovalent inter/intramolecular interactions, including halogen bonding, hydrogen bonding and π–π stacking. As such, the synergistic effect can not only restrict the molecular motion to suppress the non-radiative loss, but enhance the ISC rate to boost triplet excitons, enabling highly efficient RTP with ultralong lifetime. Moreover, the much more intensive heavy-atom effect obtained by extending the chloride RTP system to bromide is found to be unfavourable for the RTP efficiency. Finally, multifunctional applications based on these materials are demonstrated.

## Results and discussion

**Fig. 1:**
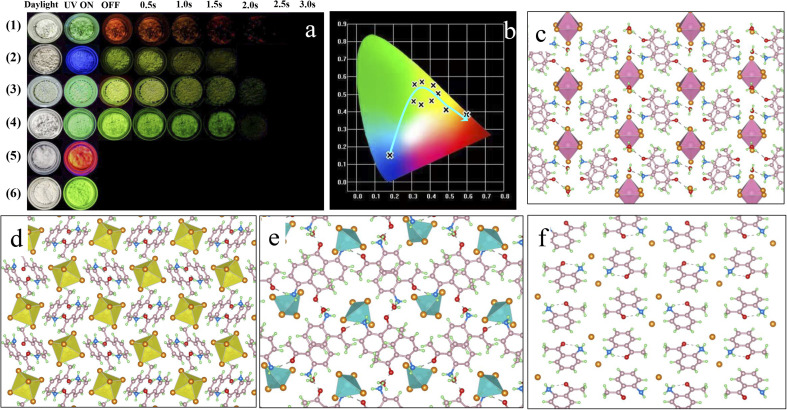
(a) Optical photographs of ACl (1), A_2_H_3_OInCl_6_·H_2_O (2), A_2_SnCl_6_ (3), A_2_ZnCl_4_·H_2_O (4), ABr (5) and A_2_ZnBr_4_·H_2_O (6) crystals under natural light, and under 365 nm UV light and UV off conditions. (b) Corresponding CIE chromaticity coordinates obtained from the photoluminescence spectra of the different materials in Fig. 1a. (c–f) Crystal structures of A_2_H_3_OInCl_6_·H_2_O (2), A_2_SnCl_6_ (3), A_2_ZnCl_4_·H_2_O (4) and ACl (1) viewed along the *a* axis.

To construct a rigid structure and improve the SOC for a highly efficient RTP system, 2-aminoacetophenone, various environmentally friendly metal ions (In^3+^, Sn^4+^ and Zn^2+^) and halide ions (Cl^−^ and Br^−^) were selected for the assembly of 0D metal-halide hybrids (Fig. S1†). As expected, efficient and tunable RTP systems were achieved based on the resultant products (Fig. 1a and b). Single-crystal structure analysis reveals that inorganic metal-halide units (anion groups) are separated from each other by the organic cation (protonated 2-aminoacetophenone, abbreviated as “A”), forming the typical 0D structure at the molecular level (Fig. 1c–e and S2a–c†). This is due to the fact that the large organic cation can act as a spacer, thereby favoring the formation of 0D structures. Detailed analysis reveals that A_2_H_3_OInCl_6_·H_2_O adopts the triclinic space group *P*1̄ (Table S1†), with one In^3+^ coordinated with six Cl^−^ to form an isolated (InCl_6_)^3−^ octahedron. A_2_SnCl_6_ and A_2_ZnCl_4_·H_2_O adopt monoclinic space groups (*P*2_1_/*n* and *P*2_1_/*c*), and are constructed by an isolated (SnCl_6_)^3−^ octahedron and (ZnCl_4_)^2−^ tetrahedron, respectively (Table S1†). The nearest In⋯In, Sn⋯Sn and Zn⋯Zn distances are 12.08, 9.75 and 8.98 Å, respectively, ruling out the interaction of adjacent inorganic units. 2-Aminoacetophenone hydrochloride (ACl) was prepared for comparison, and also adopted a 0D structure with the larger Cl^−^ filling into the gaps between the cations (Fig. 1f, S2d and Table S2†). The reliability of the structures was further validated by a series of characterizations, as displayed in (Fig. S3–5†). For metal-halide hybrids, the inorganic metal-halide units can coordinate strongly with the organic cation to form a long-range ordered arrangement *via* cation–anion electrostatic interactions, which enables materials with improved rigid structure and thermal stability, as confirmed in Fig. S6.† Due to the presence of the π-conjugated system and the heteroatoms N and O (containing *n*-antibonding orbital electrons) in the organic cation, inter/intramolecular interactions such as halogen (N–H⋯Cl, C–H⋯Cl) bonding and hydrogen (N–H⋯O, O–H⋯O) bonding are expected to form. Furthermore, the molecular packing was modulated significantly in the presence of the metal. It should be noted that both A_2_H_3_OInCl_6_·H_2_O and A_2_ZnCl_4_·H_2_O contain bound water (H_2_O) that will form multiple hydrogen bonds to enforce the intermolecular interaction. As such, multiple inter/intramolecular interactions and π–π stacking might lead to a rigid structure that restricts molecular rotation and improves the photophysical properties, thus decreasing non-radiative transition to give efficient RTP with an ultralong lifetime.

**Fig. 2:**
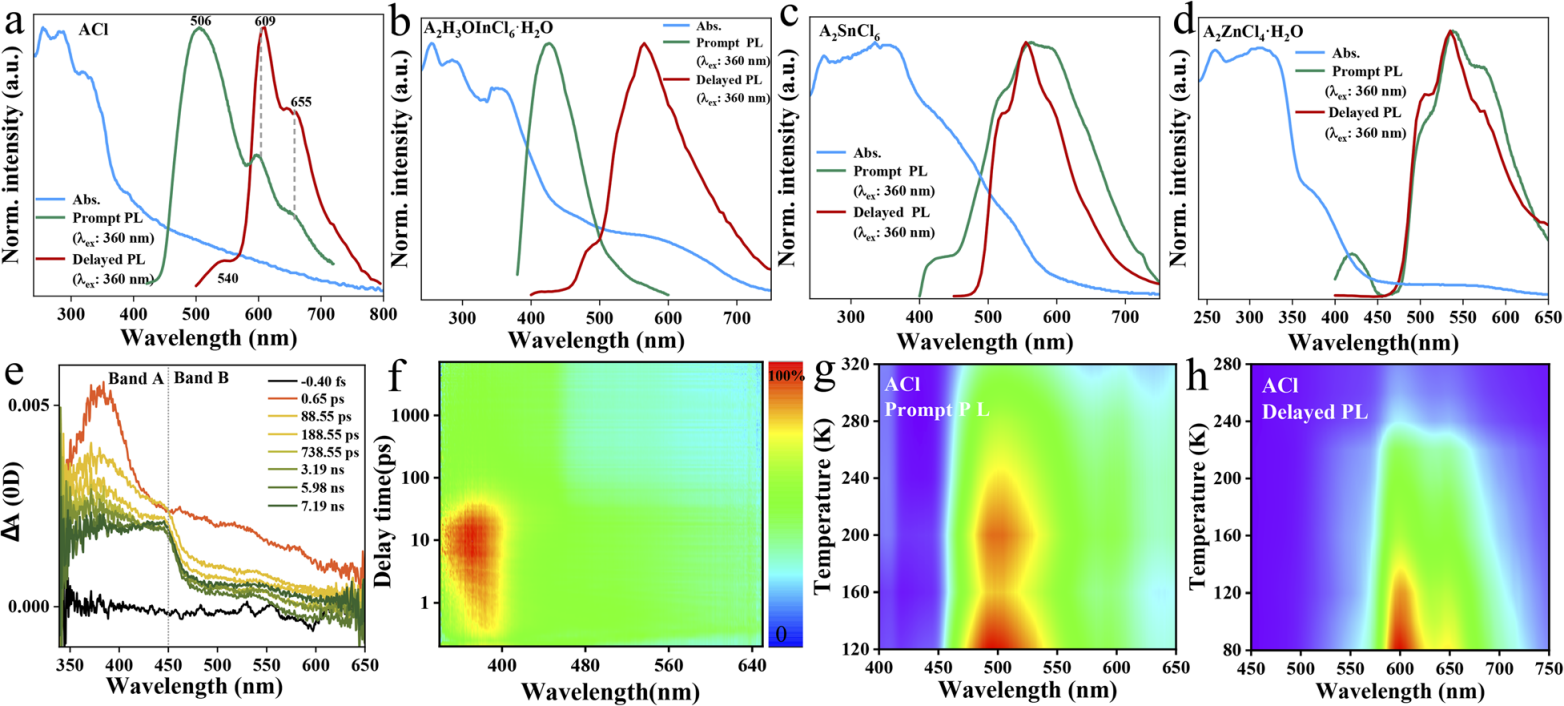
Photophysical investigations. UV-vis absorption, prompt and delayed photoluminescence spectra for bulk ACl (a), A_2_H_3_OInCl_6_·H_2_O (b), A_2_SnCl_6_ (c) and A_2_ZnCl_4_·H_2_O (d), recorded at RT. (e) and (f) Are fs-TA spectra for the A_2_ZnCl_4_·H_2_O as a function of wavelength and delay time upon photoexcitation at 330 nm at RT. (g) Temperature-dependent prompt PL spectra of ACl. (h) Temperature-dependent delayed PL spectra of ACl.

The photophysical performance of ACl and the metal-halide hybrids were first studied in both prompt and delayed modes at room temperature. The prompt PL spectrum of the metal-free ACl exhibits typical three-band emission, one with higher peak intensity located at 506 nm and two with weaker intensity at 609 and 655 nm, respectively (Fig. 2a). Its delayed PL spectrum almost perfectly overlaps the long-wavelength region of the prompt one, indicating that ACl exhibits both fluorescence and RTP characteristics. Under a 365 nm UV lamp, ACl crystals exhibit light-green emission, and the corresponding afterglow emission (UV off) is red and lasts for over 2.0 s (Fig. 1a). Subsequent temperature-dependent spectra analysis of ACl revealed that both its prompt and delayed spectra are enhanced with decreasing temperature, due to the suppression of molecular motion and reduced nonradiative rate at low temperature (Fig. 2g and h), which is further supported by the increased lifetime at low temperature (Fig. S7†). With the introduction of metals into ACl, the photophysical performance of the resultant A_2_H_3_OInCl_6_·H_2_O, A_2_SnCl_6_ and A_2_ZnCl_4_·H_2_O show the following features (Fig. 2b–d): (1) they all exhibit both fluorescence and RTP characteristics; (2) their prompt and delayed spectra are blue-shifted compared with that of ACl, and their Δ*E*_st_ (the singlet and triplet energy gap) values are all over 0.36 eV, eliminating the possibility of TADF (thermally activated delayed fluorescence),^49^ (3) the fluorescence/phosphorescence intensity ratio of the three metal-halide hybrids varies significantly due to the strong competition between the fluorescence and RTP, indicating the influence of the structure on the photophysical performance. Thus, it is easy to conclude that emissions from the three metal-halide hybrids originate from the organic component, and the spectral variation is induced by the inorganic metal-halide units. Furthermore, emission from permanent defects is also eliminated because they all exhibit linear dependence on the excitation power (Fig. S8†). For permanent lattice defects, the concentration of impurities is finite, meaning that the PL intensity would be saturated at high excitation power density. As a result, A_2_H_3_OInCl_6_·H_2_O exhibits deep-blue fluorescence (425 nm) under UV light, while A_2_SnCl_6_ and A_2_ZnCl_4_·H_2_O show light-green fluorescence similar to that of ACl (Fig. 1a). Due to the blue shift of the delayed spectra, A_2_H_3_OInCl_6_·H_2_O and A_2_SnCl_6_ exhibit Kelly afterglow emission, but A_2_ZnCl_4_·H_2_O presents green afterglow emission lasting 2.0 s (UV off). Obviously, tunable fluorescence and RTP (Fig. 1a, b and Table S3†) were realized by assembling the organic cation and different metals. Crucially, the QY_phos._ value of ACl recorded under ambient conditions at room temperature was only 2.63% (Table S4†), while those of the three metal-halide hybrids were 7.88% (A_2_H_3_OInCl_6_·H_2_O), 24.54% (A_2_SnCl_6_) and 56.56% (A_2_ZnCl_4_·H_2_O), respectively. The QY_phos._ of A_2_ZnCl_4_·H_2_O exceeds that of the vast majority of other reported solid-state RTP materials with ultralong lifetime (*τ* > 100 ms) (Table S5†). The enhanced QY_phos._ is directly associated with the enhanced ISC rate (*K*_isc_). For example, the *K*_isc_ of A_2_ZnCl_4_·H_2_O is 3.4 × 10^8^ s^−1^, which is more than 40 times that of ACl (Table S6†).

Transient PL and time-resolved spectra (TRES) characterizations were further carried out. As presented in Fig. S9,† the fluorescence lifetimes monitored at the high-energy emission (*i.e.*, 506 nm for ACl, 425 nm for A_2_H_3_OInCl_6_·H_2_O, 418 nm for A_2_SnCl_6_ and 415 nm for A_2_ZnCl_4_·H_2_O) range from 1.66 to 11.67 ns, while their corresponding RTP lifetimes measured at the low-energy emission (*i.e.*, 610 nm for ACl, 560 nm for A_2_H_3_OInCl_6_·H_2_O, 550 nm for A_2_SnCl_6_ and 535 nm for A_2_ZnCl_4_·H_2_O) range from 125 to 302 ms (Fig. S10†). The nanosecond and millisecond lifetimes further support that they exhibit both fluorescence and RTP characteristics. Compared with the RTP lifetime of the metal-free ACl (302 ms), the decreased lifetimes of the three metal-halide hybrids are associated with enhanced QY_phos._ (competition between lifetime and QY_phos._). Additionally, taking A_2_ZnCl_4_·H_2_O and ACl as representatives, their TRES also validate the ultralong RTP properties, and A_2_ZnCl_4_·H_2_O also exhibits a shortened RTP lifetime (Fig. S11†). The charge carrier dynamics of A_2_ZnCl_4_·H_2_O were further studied using femtosecond transient absorption (fs-TA) spectra. Under excitation at 330 nm with a low pulse energy of 0.5 μJ cm^−2^ pulse^−1^, hot charge carriers are injected into A_2_ZnCl_4_·H_2_O, followed by cooling process to the band edge. As presented in Fig. 2e and f, a broadband photoinduced absorption below the bandgap extending across the visible spectrum was observed, wherein band A (350–450 nm) and band B (450–650 nm) develop in sequence. The intense absorption of band A is in accordance with the corresponding UV-vis absorption spectrum (Fig. 2d), which might be associated with the excited singlet state S_1_ due to the ultrafast process. Band B is attributed to the excited triplet state T_1_ due to the fast ISC process from the S_1_ state.

As exhibited in Fig. S12,† both the prompt and delayed spectra of A_2_H_3_OInCl_6_·H_2_O, A_2_SnCl_6_ and A_2_ZnCl_4_·H_2_O show an identical trend in which the emission intensity increases with decreasing the temperature to 80 K. This is due to the fact that cryogenic conditions could suppress the non-radiative transition through the efficient confinement of molecular motion at the atomic level,^50^ which is further supported by the increased lifetime at low temperature (Fig. S13†). Generally, most organic fluorescent molecules show short-lived singlet exciton emission. Only a small fraction can break the spin-forbidden ISC process, enabling an RTP process with a lifetime of <10 ms. Herein, the three metal-halide hybrids all exhibited enhanced RTP properties with lifetimes over 100 ms. We consider that the strong cation–anion electrostatic interaction, in combination with the multiple inter/intramolecular interactions, such as hydrogen bonding and halogen bonding, might provide a rigid structure to suppress the non-radiative loss, leading to efficient RTP properties. To provide deeper insight into the underlying mechanism, single-crystal analysis was further conducted.

The key point for realizing highly efficient RTP is to enhance the SOC and promote the ISC process. It is accepted that heavy-atom and strong halogen bonding effects in a rigid structure can prevent molecular motions and stabilize triplet excitons, which will reinforce the ISC process, facilitating the generation of efficient RTP.^49,51,52^ For the three 0D metal-halide hybrids, the heavy metal and Cl^−^ ions can induce a strong heavy-atom effect and halogen bonding interactions, affording a more rigid structure compared with ACl. Halogen bonding is a type of noncovalent interaction, which is usually intentionally introduced into pure organic materials to promote SOC, thereby facilitating the ISC process.^29,53^ As illustrated in Fig. S14,† multiple halogen bonding interactions (N–H⋯Cl and C–H⋯Cl) exist in A_2_H_3_OInCl_6_·H_2_O, A_2_SnCl_6_ and A_2_ZnCl_4_·H_2_O, with the bond distances ranging from 2.27–3.84 Å. Compared with ACl (Fig. S15a†), more Cl^−^ ions are needed to coordinate with the metal to form inorganic metal-halide units, thereby leading to strong halogen bonding interactions. Hirshfeld surface analyses of the organic cations displayed in Fig. S16–19† reveal that the proportions of H–Cl are 50.5%, 55.3% and 40.3% for A_2_H_3_OInCl_6_·H_2_O, A_2_SnCl_6_ and A_2_ZnCl_4_·H_2_O, respectively, which are much higher than that of ACl (26.4%), verifying the enhanced halogen bonding interactions in the presence of metals. Thus, strong halogen bonding and the heavy-atom effect endow the three 0D metal-halide hybrids with rigid structures, which can suppress the molecular motion and promote the ISC process. As a result, many excitons are generated to reduce the non-radiative rate, thereby leading to efficient RTP.

**Fig. 3:**
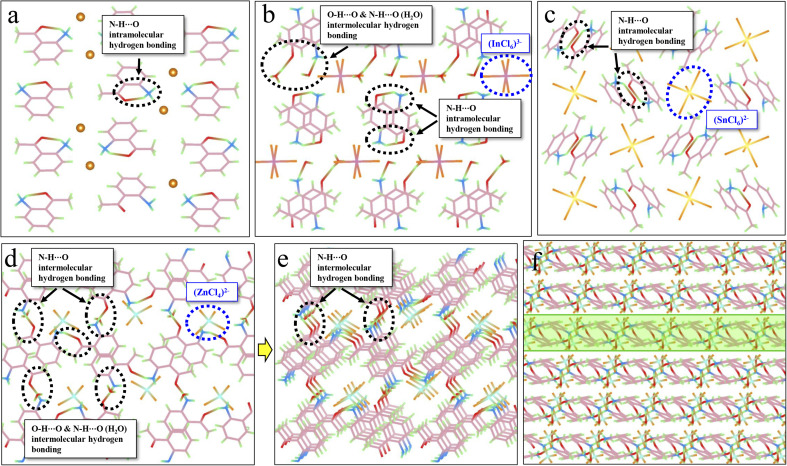
Investigation of the relationship between hydrogen bonding and efficient RTP. (a) Intramolecular hydrogen bonds (N–H⋯O) (circled by black dashed lines) in ACl. (b) Intermolecular hydrogen bonds (O–H⋯O and N–H⋯O) in A_2_H_3_OInCl_6_·H_2_O. The blue circle shows the [InCl_6_]^3−^ metal-halide unit. (c) Intramolecular hydrogen bonds (N–H⋯O) in A_2_SnCl_6_. The blue circle shows the [SnCl_6_]^3−^ metal-halide unit. (d and e) Intermolecular hydrogen bonds (N–H⋯O) in A_2_ZnCl_4_·H_2_O. The blue circle shows the [ZnCl_4_]^2−^ metal-halide unit. (f) Intermolecular hydrogen bonds forming a rigid 2D network in A_2_ZnCl_4_·H_2_O.

Hydrogen bonding, another crucial intermolecular interaction, can confine the molecular motions in molecular crystals, creating a rigid environment. Hydrogen bonds can be divided into strong (such as N–H⋯O, O–H⋯O) and weak types (such as C–H⋯O, C–H⋯π). As displayed in Fig. 3a, intramolecular hydrogen bonds (N–H⋯O) are observed in ACl, which might contribute to the corresponding red RTP (QY_phos._: 2.63%). The formation of metal-halide hybrids here will further enforce the hydrogen-bonding interaction (Table S7†). For example, the presence of protonated H_2_O in A_2_H_3_OInCl_6_·H_2_O can result in the formation of both intramolecular (N–H⋯O) and intermolecular (O–H⋯O) hydrogen bonds (Fig. 3b), which might contribute to the three-fold increase in QY_phos._ (7.88%). For A_2_SnCl_6_, abundant intramolecular hydrogen bonds (N–H⋯O) are also observed (Fig. 3c). Crucially, abundant intermolecular (N–H⋯O) hydrogen bonds exist in A_2_ZnCl_4_·H_2_O due to the presence of bound water (H_2_O) (Fig. 3d, e and S20†). These strong hydrogen bonds result in the formation of a rigid 2D network (Fig. 3f), which effectively suppresses molecular motions and non-radiative dissipations, promoting the QY_phos._ to 56.56%. The more rigid structures and the multiple inter/intramolecular interactions in the three metal-halide hybrids are further validated by the blue shift of the delayed spectra (Fig. 2b–d), compared with that of ACl (Fig. 2a). This is due to the fact that molecules in the rigid crystal should have relatively higher potential energy.^54,55^

To probe the mechanism of the ultralong lifetime, the molecular packing mode was further investigated for its significant influence on the stabilization of triplet excitons. To achieve ultralong RTP, a stable excited triplet state is needed, which could be realized by either restraining the molecular motion to reduce the non-radiative decay rate, or alternatively, increasing the potential energy of S_1_ (*i.e.*, increasing the Δ*E*_st_). The crystal structure analysis presented in Fig. S21† reveals that all the organic cations (*i.e.*, phenyl rings) in metal-free ACl display long-term π–π stacking in the form of H-aggregation. This stacking can result in an efficient electronic communication between the molecular orbitals of a material, which can stabilize the triplet excitons through enhancing the ISC process.^37,54^ Therefore, an ultralong RTP lifetime of 302 ms was recorded for ACl. For the three metal-halide hybrids, a portion of the organic cations participate in the coupling effect (Fig. S22†), indicating a decrease in the π–π stacking interaction compared with ACl (Table S8†). Although this can reduce the lifetime of the metal-halide hybrids, the enhanced structural rigidity and inter/intramolecular interactions will promote the stabilization of triplet excitons. Therefore, the three metal-halide hybrids show relatively long lifetimes in the range of 125–159 ms. In short, the presence of π–π stacking, rigid structure and inter/intramolecular interactions not only lock the molecular conformation and restrict the vibrational and rotational relaxation, thus suppressing non-radiative deactivation, but also provide stable triplet states, resulting in relatively long RTP lifetimes for the three metal-halide hybrids.

**Fig. 4:**
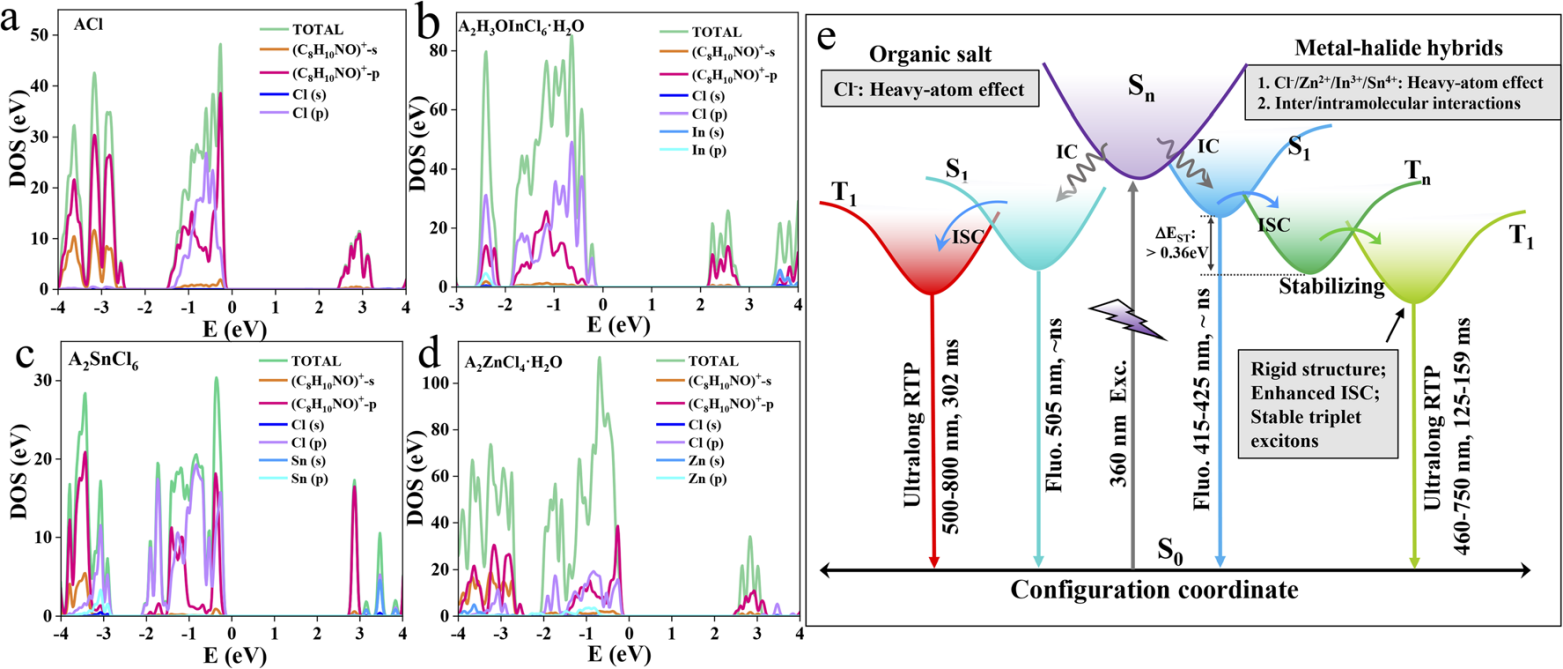
Theoretical calculations. PDOS plots of ACl (a), A_2_H_3_OInCl_6_·H_2_O (b), A_2_SnCl_6_ (c) and A_2_ZnCl_4_·H_2_O (d). (e) Schematic diagram of the photophysical mechanisms of the organic salt and metal-halide hybrids with tunable excited energy level and decay dynamics.

Theoretical calculations were further carried out to probe the band/electronic structures of the materials. Frontier orbital analysis from the projected density of state (PDOS) (Fig. 4) and electron-density distribution (Fig. S23–26†) reveal that the highest occupied molecular orbital (HOMO) and the lowest unoccupied molecular orbital (LUMO) of the three metal-halide hybrids are predominantly localized on the organic cations, suggesting that the photophysical processes essentially originate from the organic component. This is further validated by the fact that ACl and the metal-halide hybrids show quite similar UV-vis absorption spectra in dilute dichloromethane solution (Fig. S27†). By further analysing the PDOS, we find that both the HOMO and LUMO of ACl are predominantly composed of C_8_H_10_NO^+^-p orbitals. The HOMOs of A_2_H_3_OInCl_6_·H_2_O, A_2_SnCl_6_ and A_2_ZnCl_4_·H_2_O mainly consist of C_8_H_10_NO^+^ -p and Cl-3p orbitals, and their LUMOs are mainly composed of C_8_H_10_NO^+^-p, with a slight contribution from C_8_H_10_NO^+^-s. This confirms that the heavy-atom effect induced by the Cl atom is strong enough to affect the photophysical properties of the organic cation in metal-halide hybrids. Furthermore, the band structure calculation shows the following features (Fig. S28†): (1) the calculated band-gaps of the three metal-halide hybrids are close to the experimental results obtained from the UV-vis absorption edge; (2) they all exhibit almost flat bands, indicating that the excited excitons are highly localized within the organic component, facilitating the directly radiative recombination. Thereby, the multiple intermolecular interactions, a strong heavy-atom effect and strong confinement effect in rigid 0D structures endow the metal-halide hybrids with highly stable and localized triplet excitons, facilitating highly efficient RTP with an ultralong lifetime, as schematically illustrated in Fig. 4e. Therefore, our strategy can be adopted to construct long-lasting afterglow materials with high PLQY.

### Extending the scope of the RTP system

We further extended the scope of the RTP systems to the bromides. As shown in Fig. S29 and S30,† ABr and A_2_ZnBr_4_·H_2_O exhibit a similar structure to ACl and A_2_ZnCl_4_·H_2_O, respectively, but their photoluminescence properties are significantly different (Fig. S31a and S32a†). For example, ABr and A_2_ZnBr_4_·H_2_O exhibit red (665 nm) and green (540 nm) light (Fig. 1a), respectively, without visible afterglow emission being observed. As displayed in Fig. S31a,† the prompt and delayed spectra of ABr are different, eliminating its TADF property. Its RTP lifetime is only 10.54 ms (Fig. S31b†). The temperature-dependent delayed spectra of ABr shown in Fig. S31c† present the enhancement of the delayed spectrum with decreased temperature, suggesting the suppression of molecular motion and reduced nonradiative rate at low temperature. The RTP lifetime of A_2_ZnBr_4_·H_2_O is 12.03 ms (Fig. S32b†), which is much shorter than that of its chloride counterpart (A_2_ZnCl_4_·H_2_O). The significantly shortened lifetimes of ABr and A_2_ZnBr_4_·H_2_O might be due to the fact that Br can induce a much more intense heavy-atom effect that can accelerate the decay rates of the triplet state, leading to short lifetimes.^56^ Moreover, structure analysis unveils that there are some minor differences between the bromide and chloride counterparts. As listed in Tables S1 and S2,† the lattice constants of ABr and A_2_ZnBr_4_·H_2_O are slightly larger than those of ACl and A_2_ZnCl_4_·H_2_O, resulting in the larger cell volumes of ABr (853.5 *vs.* 815.9 Å^3^) and A_2_ZnBr_4_·H_2_O (2237.8 *vs.* 2124.7 Å^3^) and higher crystal densities of ABr (1.68 *vs.* 1.40 g cm^−3^) and A_2_ZnBr_4_·H_2_O (2.00 *vs.* 1.56 g cm^−3^). As such, the crystals of ABr and A_2_ZnBr_4_·H_2_O will adopt loose packing styles with decreased intermolecular interactions, which will increase the non-radiative recombination and decrease the RTP efficiency. Therefore, the much more intensive heavy-atom effect and expanded lattice constant for the bromide counterparts lead to the decreased RTP lifetimes and lack of observable afterglow emission.

**Fig. 5:**
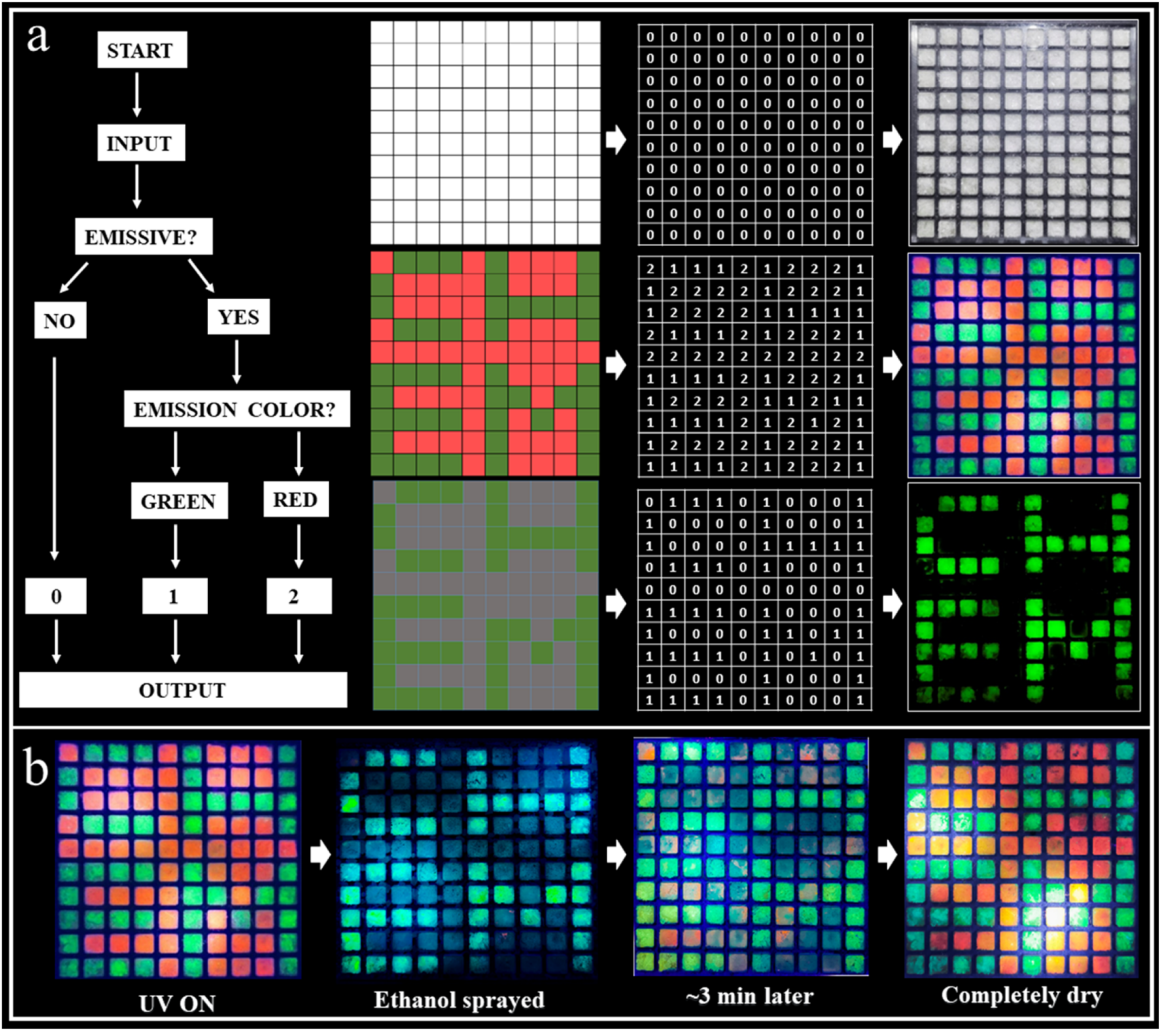
Demonstration of an efficient and tunable RTP system for multilevel information encryption or anti-counterfeiting applications. (a) Demonstration of a triple encoding model, in which ABr (red fluorescence) and A_2_ZnCl_4_·H_2_O (green fluorescence, green afterglow) were selectively deposited as a pixelated pattern (10 × 10 square matrix) on an acrylic module, showing their promising future in information encryption. (b) Process of ethanol decryption of the matrix using ABr and A_2_ZnCl_4_·H_2_O.

Information encryption and anti-counterfeiting are of great significance and have received enormous attention in this information era. Generally, single-level information encryption systems or anti-counterfeiting measures are easy to implement, but the risk of information leakage is greater. In contrast, multilevel systems have higher security, but are more challenging. Benefiting from the wide-ranging tunable fluorescence and long-lived efficient RTP characteristics of these materials, a triple encoding model was fabricated as a proof-of-concept to show their promise in information encryption. ABr (red fluorescence) and A_2_ZnCl_4_·H_2_O (green fluorescence, green afterglow) were selectively deposited as a pixelated pattern (10 × 10 square matrix) on an acrylic module (Fig. 5). In this design strategy, we define the output “0” as the lack of an emission phenomenon, and the command points “1” as red and “2” as green light. Under daylight, all squares exhibit a similar “0” output as the first disturbance information. Under a 365 nm UV lamp, all squares are emissive and give the command (red square “1” and green square “2” output) as the second invalid information. When the decryption key of the UV lamp is OFF, the red luminous squares disappear, but the green luminous points still exist. Therefore, the third correct decryption information “CHEM” is output. Interestingly, we found that ABr exhibits reversible emission switching due to the dynamic ethanol uptake/release, making it an ethanol sensor. That is, ethanol can induce the quenching of the red fluorescence for ABr due to its strong polarity, while it can be recovered to red fluorescence after drying the ethanol. As shown in Fig. 5b, when ethanol (as a key) is sprayed on the luminous squares, the red luminous squares disappear to output the green information “CHEM”. With the evaporation of the ethanol, the red luminous squares reemerge gradually, making the decryption process traceless. This unique decoding strategy that we have reported for the first time plays a multiple-protection role with an information encryption and mutual verification effect, and the security is far superior to single-level encryption and anti-counterfeiting technologies, thus showing its important application potential.

**Fig. 6:**
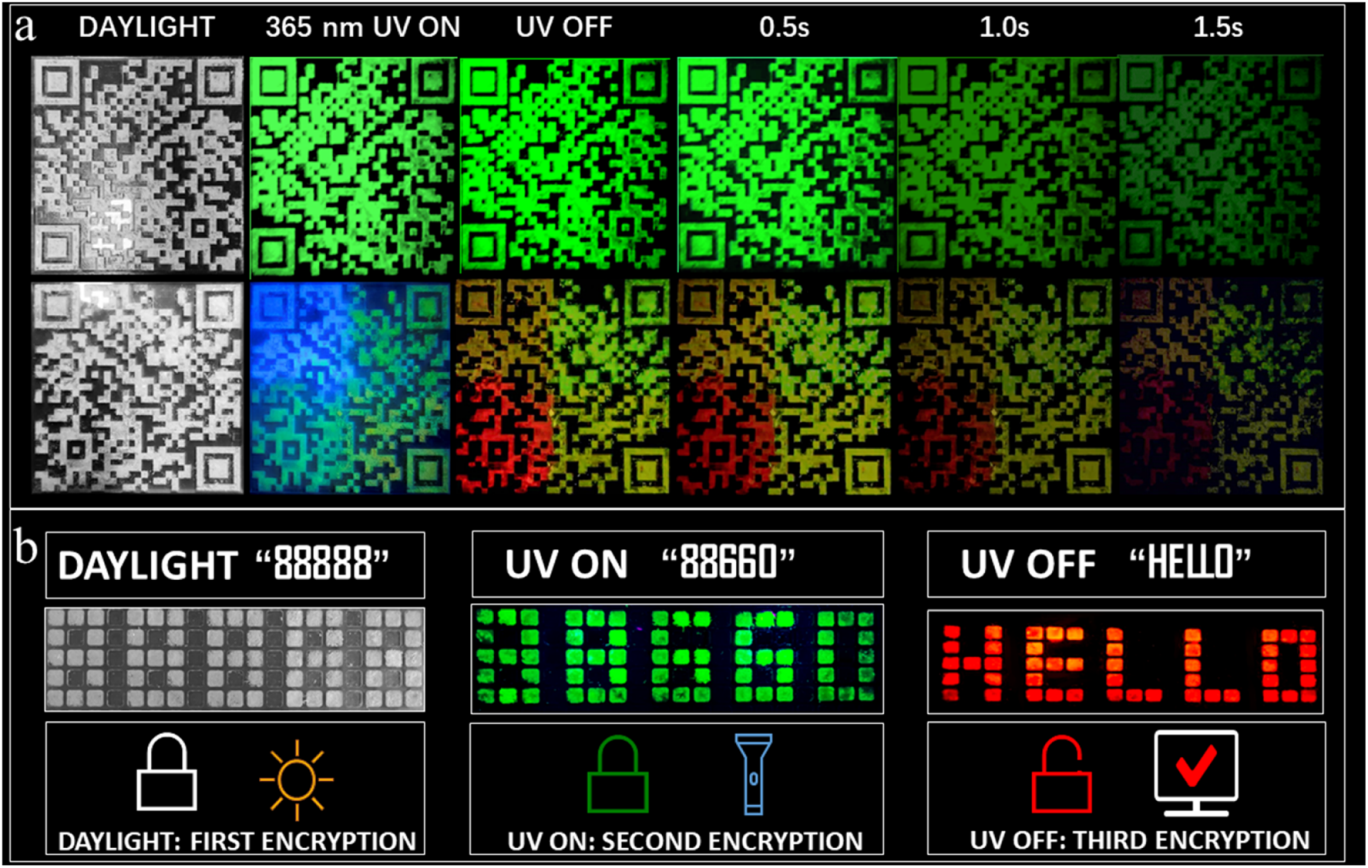
(a) Anti-counterfeiting applications using a dynamic QR code (based on A_2_ZnCl_4_·H_2_O, A_2_SnCl_6_, A_2_H_3_OInCl_6_·H_2_O and ACl) and a traditional static QR code (based on A_2_ZnCl_4_·H_2_O alone) under daylight, 365 nm UV light and UV off conditions. (b) Photos of the pattern “88 888” under different circumstances to demonstrate the visual encryption system based on ACl and A_2_ZnBr_4_·H_2_O.

Furthermore, anti-counterfeiting applications using dynamic QR codes utilizing the multicolor afterglow properties have been successfully realized. Such applications are rare among RTP systems. Fig. 6a provides a comparison between a static (composed of A_2_ZnCl_4_·H_2_O) and dynamic QR code (composed of A_2_ZnCl_4_·H_2_O, A_2_SnCl_6_, and A_2_H_3_OInCl_6_·H_2_O). Obviously, there are remarkable differences between them, especially when the UV light is OFF. The advantages of the dynamic QR code anti-counterfeiting label are as follows: (1) multiple anti-counterfeiting mechanisms with reliable functions. For example, it can provide triple anti-counterfeiting mechanisms because it includes unique digital, color and material information; (2) effectively raising the threshold for copying QR codes. This is because a dynamic QR code goes beyond the traditional QR code with a single color, forcing counterfeiters to confront increased technological costs, update equipment and raise piracy costs; (3) concise and intuitive identification. We can visually identify the anti-counterfeiting code and color information of a product, allowing the authenticity of the product to be distinguished using the naked eye. Additionally, we also designed a visual encryption system based on ACl (green fluorescence, green afterglow) and A_2_ZnBr_4_·H_2_O (green fluorescence). Both of the materials were patterned into an 88 888-shaped module. As presented in Fig. 6b, the decryption can be broken down into three steps: (1) under daylight, the figures “88 888” are observed directly; (2) under a 365 nm UV lamp, green emission with the shape “88 660” is clearly observed; (3) at a delay time of 0.1 s, the green “88 660” shape is converted into the red-emitting English letters “HELLO”. The above results suggest that the multicolor fluorescence and afterglow systems show wide forward-looking application prospects in the fields of information encryption, anti-counterfeiting and dynamic optical data storage, among others.

## Conclusions

In summary, we have reported the development of a series of 0D metal-halide hybrids featuring color-tunable, ultralong, highly efficient RTP materials based on an identical organic component. Compared to the organic chloride (ACl), which has low RTP efficiency, the three 0D metal-halide hybrids all exhibited changed crystal structures and stacking styles, leading to efficient RTP properties. Furthermore, on changing the center metal and halogen, multicolor-tunable visible range photoemission from blue (425 nm) to red (665 nm) has been achieved based on these materials. Notably, A_2_H_3_OInCl_6_·H_2_O, A_2_SnCl_6_ and A_2_ZnCl_4_·H_2_O all show enhanced RTP efficiencies and ultralong lifetimes compared with the metal-free organic chloride (ACl) due to their more rigid structures and the presence of multiple inter/intramolecular interactions and strong heavy-atom effect. Additionally, information encryption and anti-counterfeiting applications were developed based on these materials as a proof-of-concept to demonstrate their wide and forward-looking application prospects. This work not only achieved tunable RTP properties with high efficiency (QY_phos._ > 20%) and long lifetime (*τ* > 100 ms) based on organic–inorganic metal-halide hybrids, but also opens opportunities for the development of smart RTP systems for high-tech applications.

## Data availability

Experimental procedures, details of the calculations, and additional data can be found in the ESI.† Other data that support the findings of this study are available from the corresponding author upon request. Crystallographic data generated in this study for A_2_H_3_OInCl_6_·H_2_O, A_2_SnCl_6_, A_2_ZnCl_4_·H_2_O, A_2_ZnBr_4_·H_2_O, ACl and ABr have been deposited in the Cambridge Crystallographic Data Centre under accession codes CCDC 2321891, 2321896, 2321895, 2321892, 2321893 and 2321894.

## Author contributions

R. X. H. conceived the idea and supervised the project. L. Z. and K. L. L. performed the experiments and analysed the data. Y. Y. C. and M. L. assisted with spectral measurements and data analysis. Y. Y. and Y. Q. P. assisted with the single-crystal experiments. All authors contributed to the discussion and manuscript writing.

## Conflicts of interest

The authors declare no competing financial interest.

## Supplementary Material

SC-015-D4SC01630K-s001

SC-015-D4SC01630K-s002
