# Supplementary material for: High-efficiency color-tunable ultralong room-temperature phosphorescence from organic–inorganic metal halides via synergistic inter/intramolecular interactions
Source: Chem Sci. 2024 May 23;15(26):10046–55. doi: 10.1039/d4sc01630k (PMC11220578; doi:10.1039/d4sc01630k)
Supplement: SC-015-D4SC01630K-s001 [file SC-015-D4SC01630K-s001.pdf]

Electronic Supplementary Material (ESI) for Chemical Science.
This journal is © The Royal Society of Chemistry 2024

Supplementary information

High-efficiency of color-tunable ultralong room-temperature phosphorescence from organic-inorganic metal halides by synergistic inter/intramolecular interactions

Lei Zhou,‡[a] Kailei Li,‡[a] Yuanyuan Chang,[b] Yuan Yao,[a] Yuqi Peng,[a] Ming Li[a] and Rongxing He*[a]

[a]*Key Laboratory of Luminescence Analysis and Molecular Sensing (Southwest university), Ministry of Education, School of Chemistry and Chemical Engineering, Southwest University, Chongqing 400715, China*

[b]*Institute of Materials Science and Devices, School of Materials Science and Engineering, Suzhou University of Science and Technology, Suzhou 215009, China*

*Corresponding Authors: Rongxing He

E-mail addresses: herx@swu.edu.cn (R. He)

‡Authors with equal contribution to the work.

Materials: Tin (II) chloride dehydrate ($SnCl_2 \cdot 2H_2O$, 98%) and 2-Aminoacetophenone (C_8H_9NO, 99%) were purchased from Innochem (Beijing) Technology Co., LTD. Protonated 2-Aaminoacetophenone is hereinafter abbreviated as “A”. Hydrochloric acid (HCl, 37.0% in water by weight) was purchased from Chongqing Chuan Dong Chemical (Group) Co., LTD. Hydro bromic acid (HBr, AR, 40%) was purchased from Shanghai McKenlin Biochemical Technology Co., LTD. Ethyl alcohol (CH_3CH_2OH, AR) was purchased from Chongqing Titanium New Chemical Co., LTD. Indium (III) acetate ($C_2H_7InO_2$, 99%) was purchased from. Tianjin Xiensi Opdeke Technology Co., LTD. Zinc acetate dehydrate ($C_4H_6O_4Zn \cdot 2H_2O$) was purchased from Chengdu Colon Chemical Co. LTD All reagents and solvents were used without further purification. Distilled water is prepared in our lab.

Crystal growth of $A_2ZnX_4 \cdot H_2O$ (X = Cl and Br): For the synthesis of $A_2ZnX_4 \cdot H_2O$, Zinc acetate (1 mmol) and 2-Aminoacetophenone (400 μl) were dissolved in 2 mL HX (X = Cl and Br) at 80 °C with constant stirring for 0.5h to form a clear solution. Then, crystals were obtained by slow cooling to room temperature (RT) and finally dried at 45 °C in an oven.

Crystal growth of A_2SnCl_6: Crystalline A_2SnCl_6 samples were prepared via the hydrothermal method. A mixture of Tin (II) chloride dehydrate (1 mmol) with 2-Aminoacetophenone (350 μl) and 5 mL of HCl was stirred for 10 min. The mixture was then transferred to a Teflon reactor (25 mL) and heated at 130 °C for 12 h. After that, the mixture was cooled at a rate of 10 °C h-1 to RT. The colorless transparent crystals of A2SnCl6 were washed by ethanol (3 × 10 mL). Then, crystals were obtained by slow cooling to room temperature and finally dried at 45 °C in an oven.

Crystal growth of $A_2H_3OInCl_6 \cdot H_2O$: For the synthesis of $A_2H_3OInCl_6 \cdot H_2O$, Indium (III) acetate (0.5mmol) and 2-Aminoacetophenone (400 μl) were dissolved in 1 mL HCl at 100 °C with constant stirring for 1 h to form a clear solution. Then, crystals were obtained by slow cooling to RT and finally dried at 45 °C in an oven.

Crystal growth of AX (X = Cl and Br): For the synthesis of AX, 2-

Aminoacetophenone (200 ul) were dissolved in 1 mL HX (X = Cl and Br) at 100 °C with constant stirring for 10 min to form a clear solution. Then, crystals were obtained by slow cooling to room temperature and finally dried at 45 °C in an oven.

Structure (or Chemical Component) Characterizations: Single-crystal X-ray diffraction data were collected from Agilent Technologies Gemini AUltra system with Mo-Kα radiation (λ = 0.71073 Å) at RT. The structures were resolved and refined using direct methods with OLEX2. Powder X-ray diffraction (PXRD) and d were performed on an X-ray powder diffractometer (D2 Phaser, Bruker, Germany) at RT. A typical scan rate was 10 s step^{-1} with a step size of 0.02°. Thermogravimetry analysis (TGA) was carried out using a TA instrument (Q50 TGA system). The samples were heated from RT (~ 25 °C) to 800 °C with at a rate of 10 °C min^{-1} under an argon flux of 20 mL min^{-1}. XPS measurements of the samples were conducted on a Thermal K-Alpha spectrometer equipped with a monochromatic Al Kα X-ray source. Parameters of the π–π interactions and hydrogen-bond geometry in $A_2ZnCl_4{\cdot}H_2O$, $A_2ZnBr_4{\cdot}H_2O$, A_2SnCl_6, $A_2H_3OInCl_6{\cdot}H_2O$, ACl and ABr got from OLEX2.

Optical Characterizations: UV-visible absorption spectra of the materials were obtained from the Agilent Cary5000 spectrophotometer equipped with integrating sphere to exclude signal due to light scattering. $BaSO_4$ was used as a non-absorbing reflectance reference for diffuse-reflectance measurements. The photoluminescence quantum yields were measured on a calibrated integrating sphere. Steady-state RT and temperature-dependent excitation and emission spectra were recorded on a photoluminescence spectrometer (Horiba Fluorolog-3, Horiba Ltd.), and their corresponding transient photoluminescence spectra were also recorded on Fluorolog-3 with the time-correlated single-photon counting (TCSPC) mode. The photoluminescence quantum yields (PLQY) were measured on Fluorolog-3 with a calibrated integrating sphere. Time-resolved emission spectroscopy (TRES) characterization were performed on FLS 1000. In detail, a pulsed light source excites a sample, and the resulting photoluminescence attenuation is continuously recorded as a function of the emission wavelength to create a three-dimensional data that includes

photoluminescence spectra and time correlation. The FluOracle software of FLS 1000 can automatically obtain TRES spectra using TCSPC or MCS modes to measure short and long lifetimes, respectively. VPLED310 is set to a pulse width of 800 ms and 10 Hz repetition rate. Use MCS mode to obtain photoluminescence attenuation in the wavelength range of 400-700 nm with a step size of 5 nm. Fourier transform infrared spectroscopy (FTIR) was measured by the Perkin Elmer (United States), using a DTGS detector. The parameter settings: scanning range: 4000~400 cm^{-1}, resolution: 4 cm^{-1}, cumulative scanning of 4 ATR tests. Spectral analysis using the instrument's built-in Spectrum software.

Femtosecond Transient Absorption Spectroscopy Measurements: The fs-TA measurements were performed on a Helios pump-probe system (Ultrafast Systems LLC) combined with an amplified femtosecond laser system (Coherent). Optical parametric amplifier (TOPAS-800-fs) provided a 330 nm pump pulse (~ 0.5μJ/pulse, which was excited by a Ti: sapphire regenerative amplifier (Legend Elite-1K-HE; 800 nm), 35 fs, 7 mJ/pulse, 1 kHz) and seeded with a mode-locked Ti: sapphire laser system (Micra 5) and an Nd: YLF laser (EvolutIon 30) pumped. Focusing the 800 nm beams (split from the regenerative amplifier with a tiny portion, ~ 400 nJ/pulse onto a CaF_2 plate produced the white-light continuum (WLC) probe pulses (350-650nm). The pulse-to-pulse fluctuation of the WLC is corrected by a reference beam split from WLC. A motorized optical delay line was used to change the time delays (0 ~ 8 ns) between the pump and probe pulses. The instrument response function (IRF) was determined to be ~ 100 fs by a routine cross-correlation procedure. The instrument response function (IRF) was determined to be ~100 fs by a routine cross-correlation procedure. A mechanical chopper operated at a frequency of 500 Hz used to modulate the pump pulses such that the fs-TA spectra with and without the pump pulses can be recorded alternately.

Hirshfeld Surfaces and Two-Dimensional Fingerprint Plots: The intermolecular interactions for all materials in this work were investigated through Hirshfeld surface

analysis by using Crystal Explore 17.5.1 First, the CIF file was uploaded into the software. In the window of surface generation, the surface was set as "Hirshfeld", and the option "None" was chose for its property. After generating a Hirshfeld surface, "Surface property" was set as "dnorm". Further, in the window of two-dimensional fingerprint plots, the option "di vs de" was defaulted for the type. dnorm is the normalized contact distance, di is the distance from a point on the Hirshfeld surface to the nearest atom inside the surface, and de is the distance from a point on the Hirshfeld surface to the nearest atom outside the surface.

Computational Methods: All calculations were based on the density functional theory (DFT) implemented in the VASP code. The projector augmented wave (PAW) method was used with a cutoff energy of 400 eV. The electron exchange correlation effect was represented by the PerdewBurke-Ernzerhof (PBE) functional under the generalized gradient approximation (GGA). The optimization of lattice parameters was based on the experimentally measured values, while the atomic positions were optimized until the force on each atom was <0.02 eV $Å^{-1}$. The total energy was converged to 10^{-6} eV. The k-point separation was set as 0.04 $Å^{-1}$ in the Brillouin zone leading to corresponding Γ-centered k-point meshes of $A_2ZnBr_4·H_2O$: 3×1×1; A_2SnCl_6: 3× 2 × 2; ACl: 5×2×2; $A_2H_3OInCl_6·H_2O$: 3×3×1.

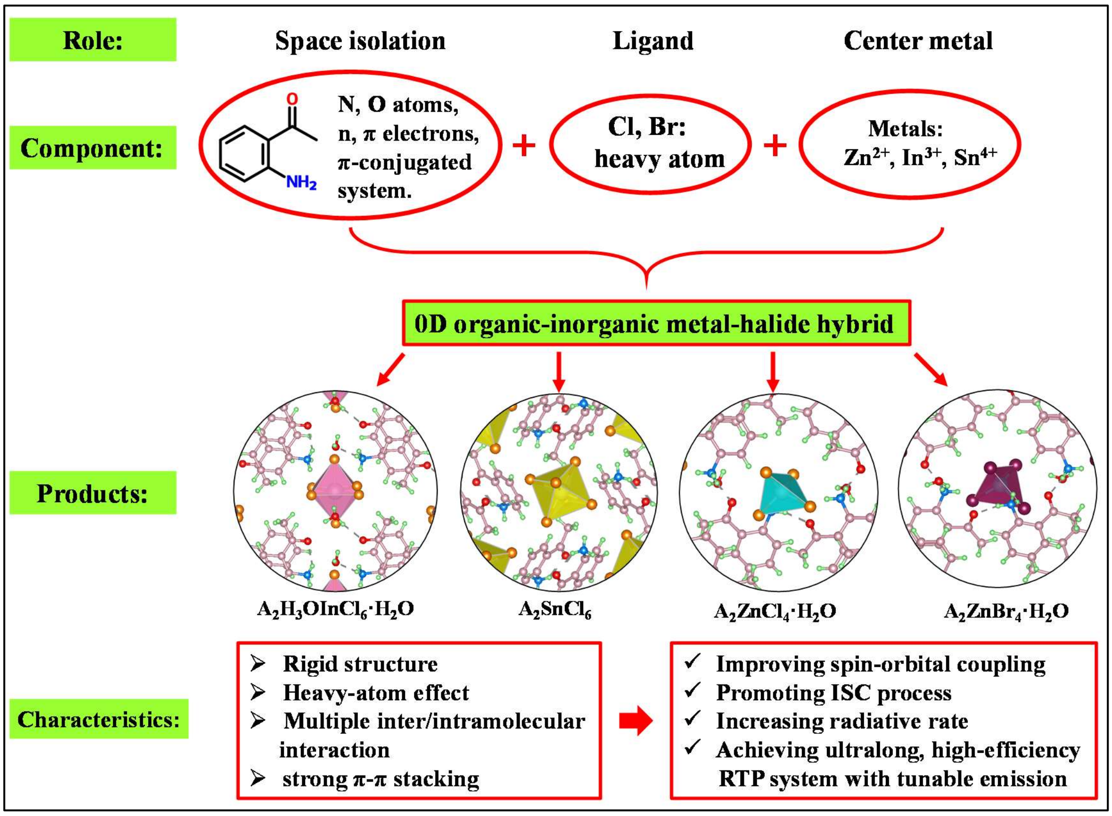

Figure S1. Schematic illustration of the strategies used to construct high-efficiency RTP system in this work.

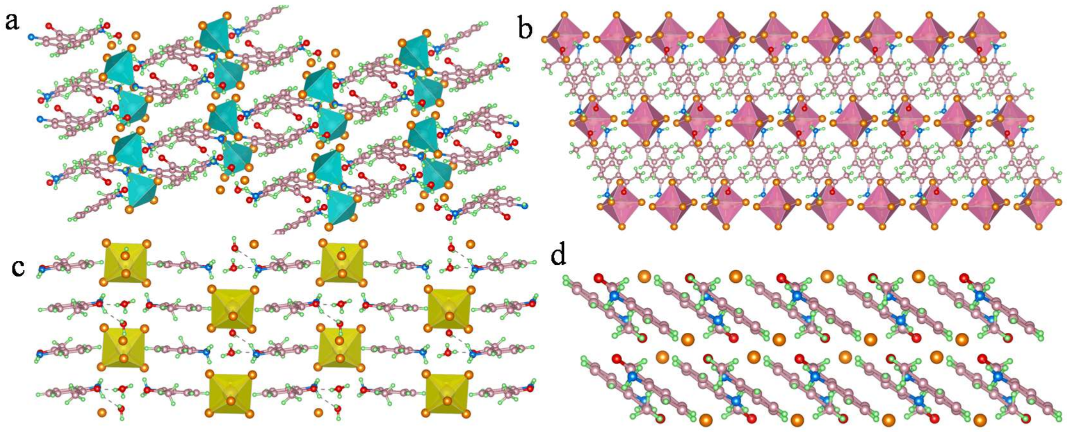

Figure S2. Detailed crystal structures viewed along b axis. (a) $A_2ZnCl_4{\cdot}H_2O$, (b) A_2SnCl_6, (c) $A_2H_3OInCl_6{\cdot}H_2O$, and (d) ACl.

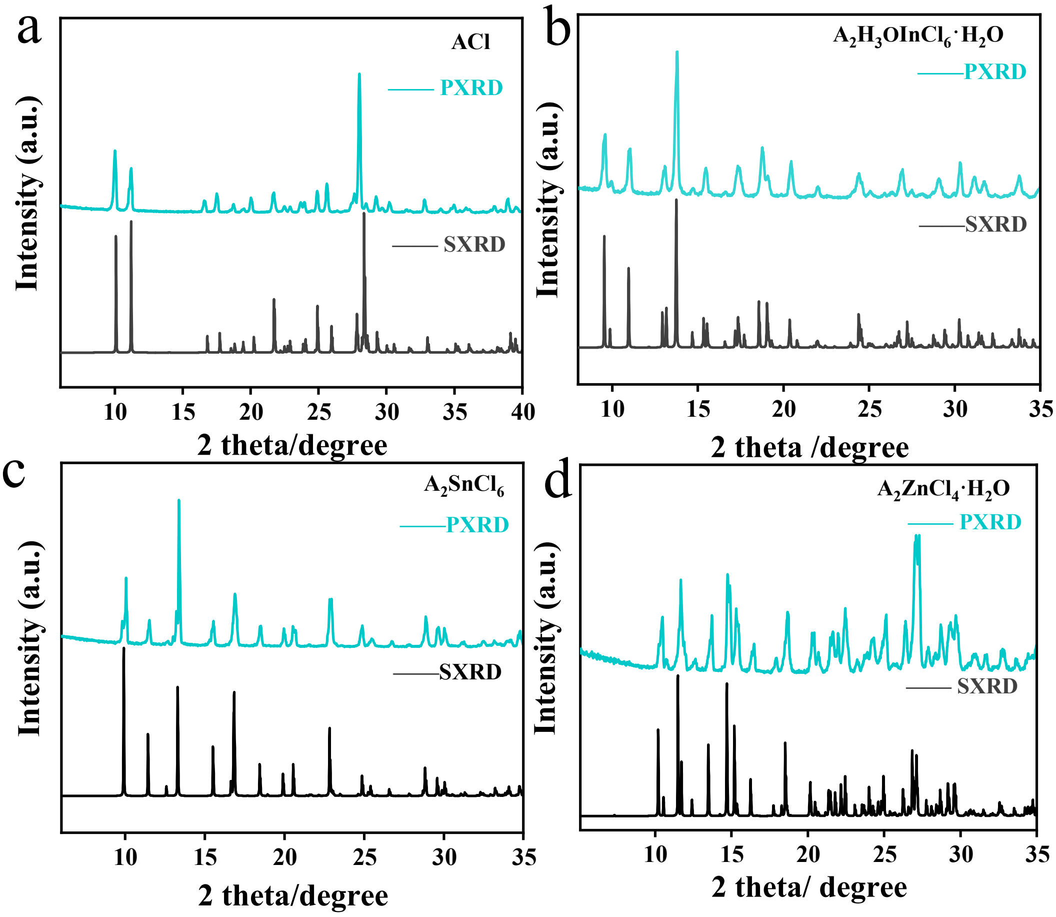

Figure S3. Comparison of the experimental PXRD and SCXRD patterns of (a) $A_2ZnCl_4{\cdot}H_2O$, (b) A_2SnCl_6, (c) $A_2H_3OInCl_6{\cdot}H_2O$ and (d) ACl.

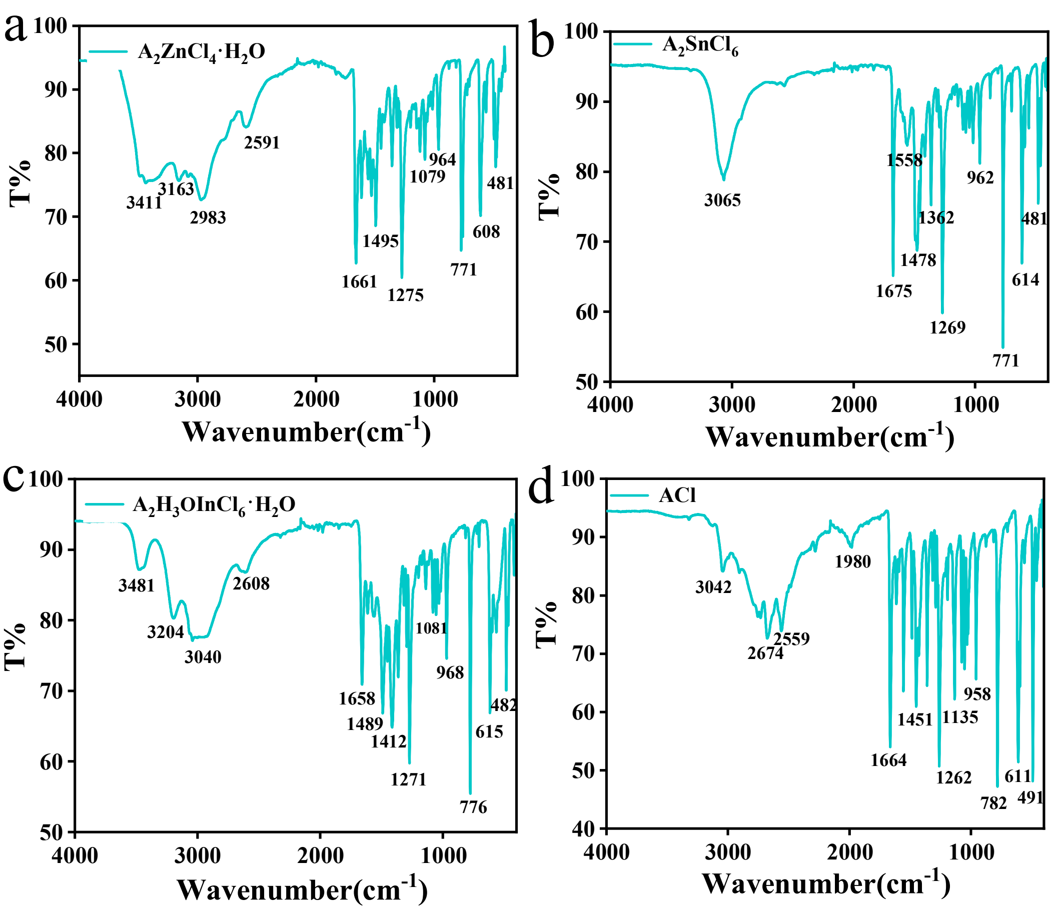

Figure S4. Fourier transform infrared spectroscopy (FTIR) of (a) $A_2ZnCl_4 \cdot H_2O$, (b) A_2SnCl_6, (c) $A_2H_3OInCl_6 \cdot H_2O$ and (d) ACl.

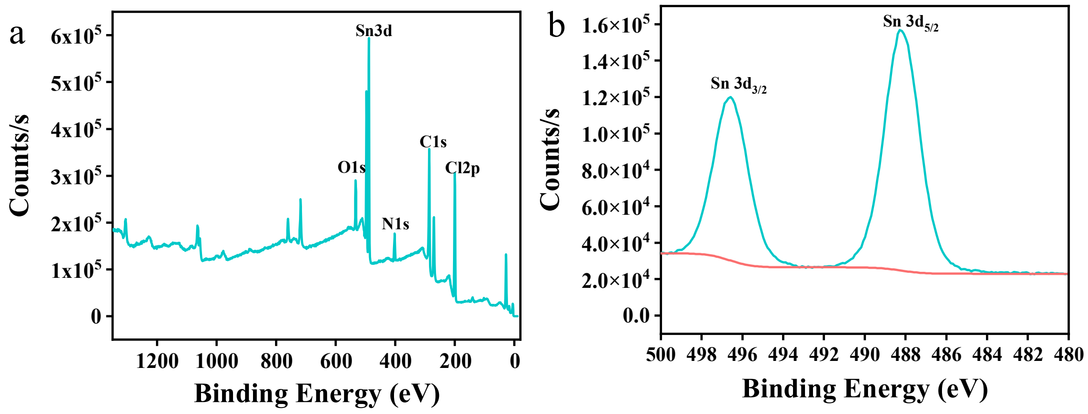

Figure S5. (a) XPS spectrum of A_2SnCl_6. (b) High-resolution XPS spectrum of Sn ion for A_2SnCl_6.

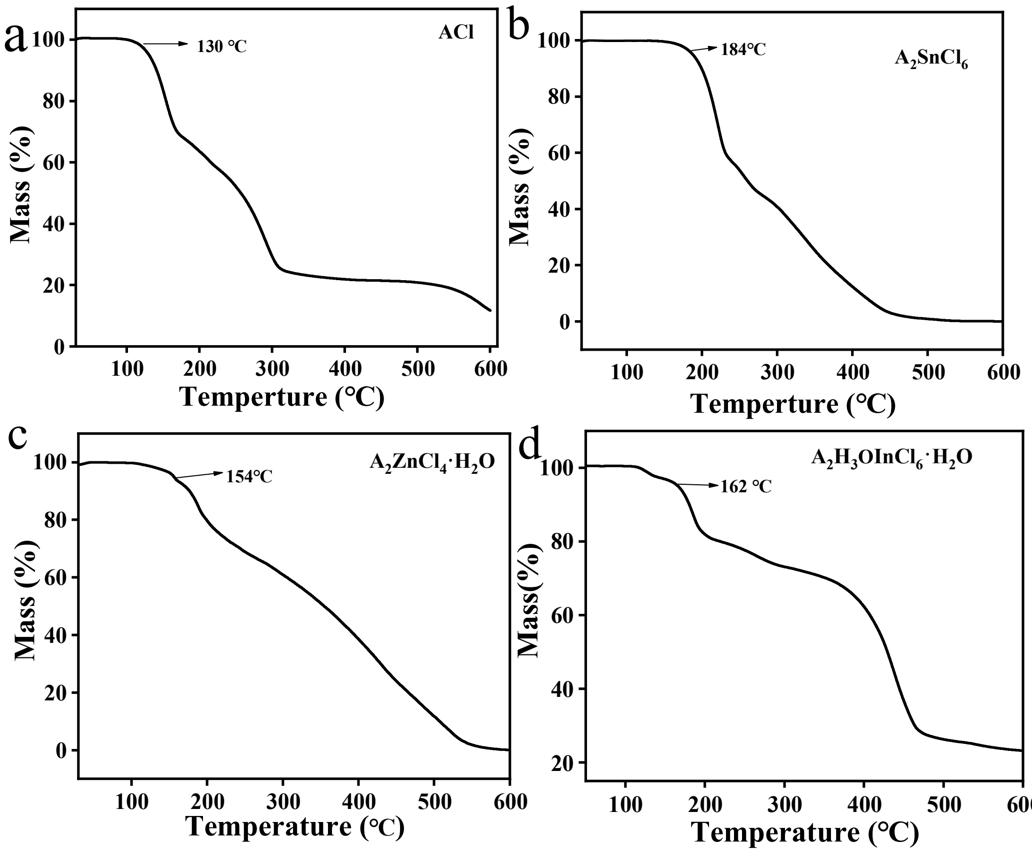

Figure S6. Thermogravimetric (TG) analysis of (a) ACl, (b) A_2SnCl_6, (c) $A_2ZnCl_4{\cdot}H_2O$ and (d) $A_2H_3OInCl_6{\cdot}H_2O$.

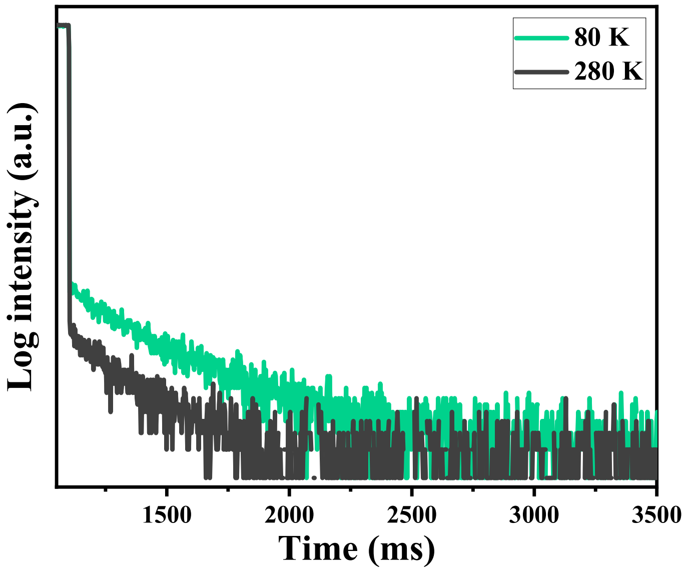

Figure S7. Transient RTP decay curve of ACl, recorded at 610 nm at 80 and 280 K.

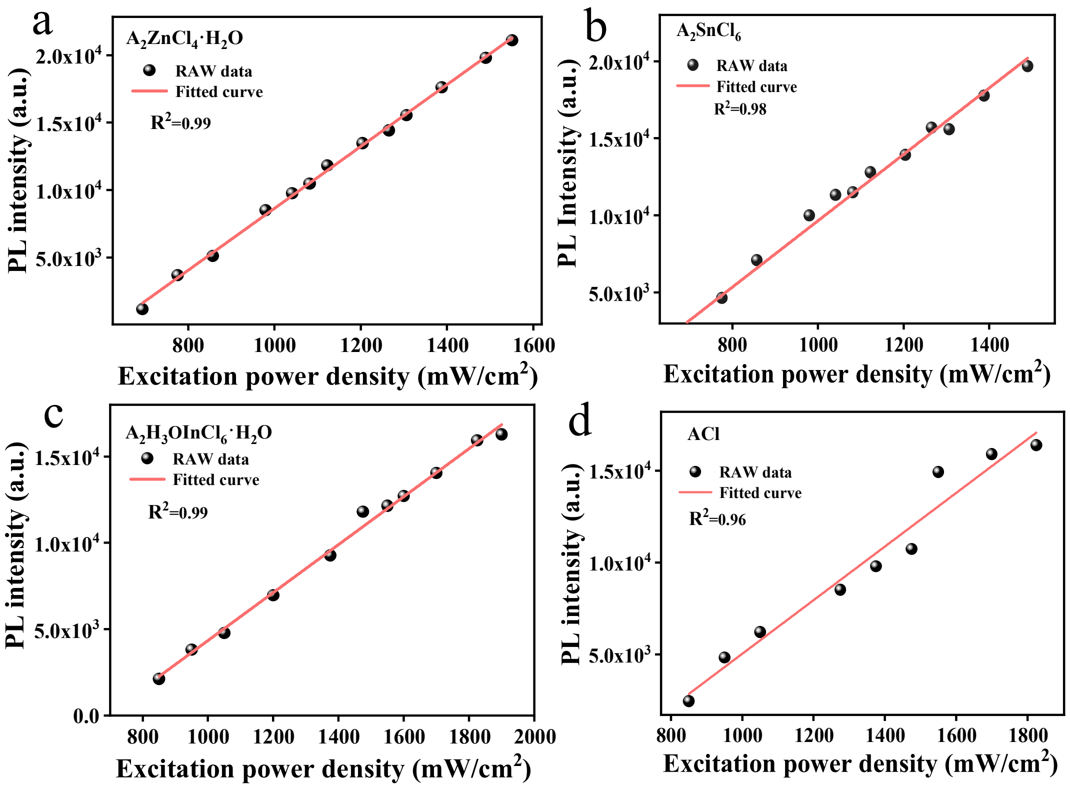

Figure S8. The plot of FL intensity of (a) $A_2ZnCl_4{\cdot}H_2O$, (b) A_2SnCl_6, (c) $A_2H_3OInCl_6{\cdot}H_2O$ and (d) ACl, as a function of excitation power density.

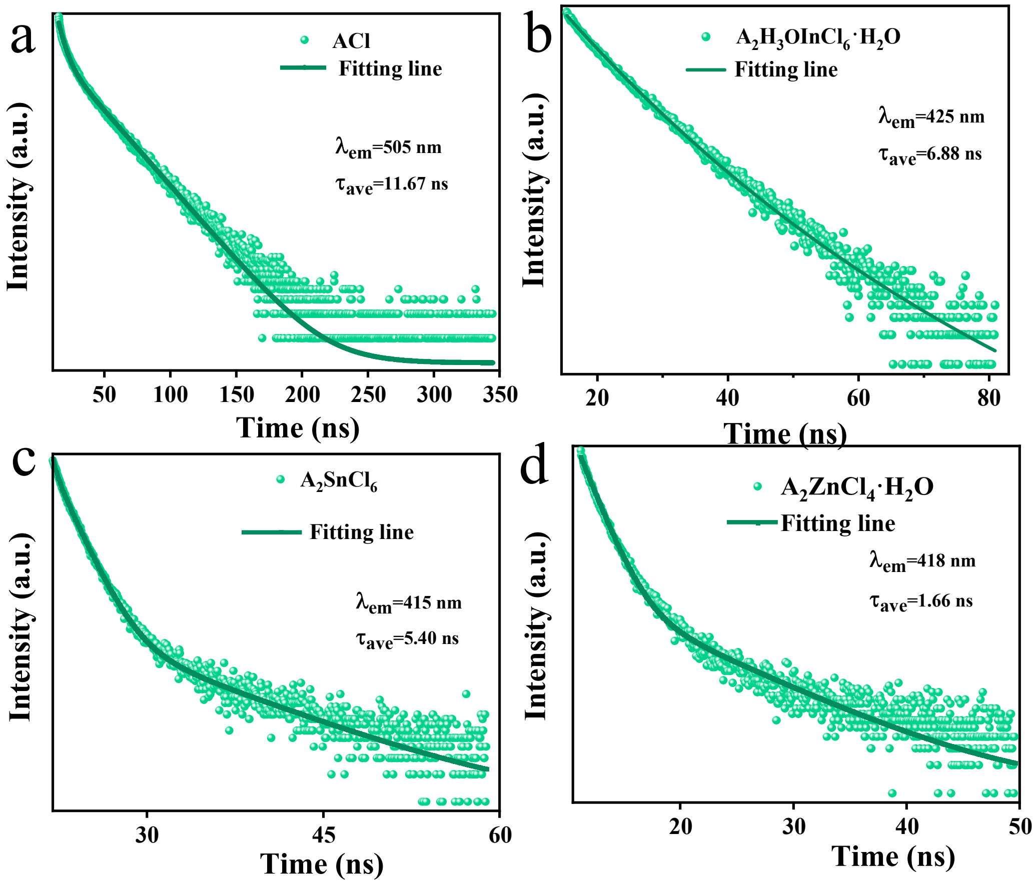

Figure S9. The ns-level singlet-state decay curves of (a) $A_2ZnCl_4{\cdot}H_2O$, (b) ACl, (c) $A_2H_3OInCl_6{\cdot}H_2O$ and (d) A_2SnCl_6, recorded at 418, 505, 425 and 415 nm at RT, respectively.

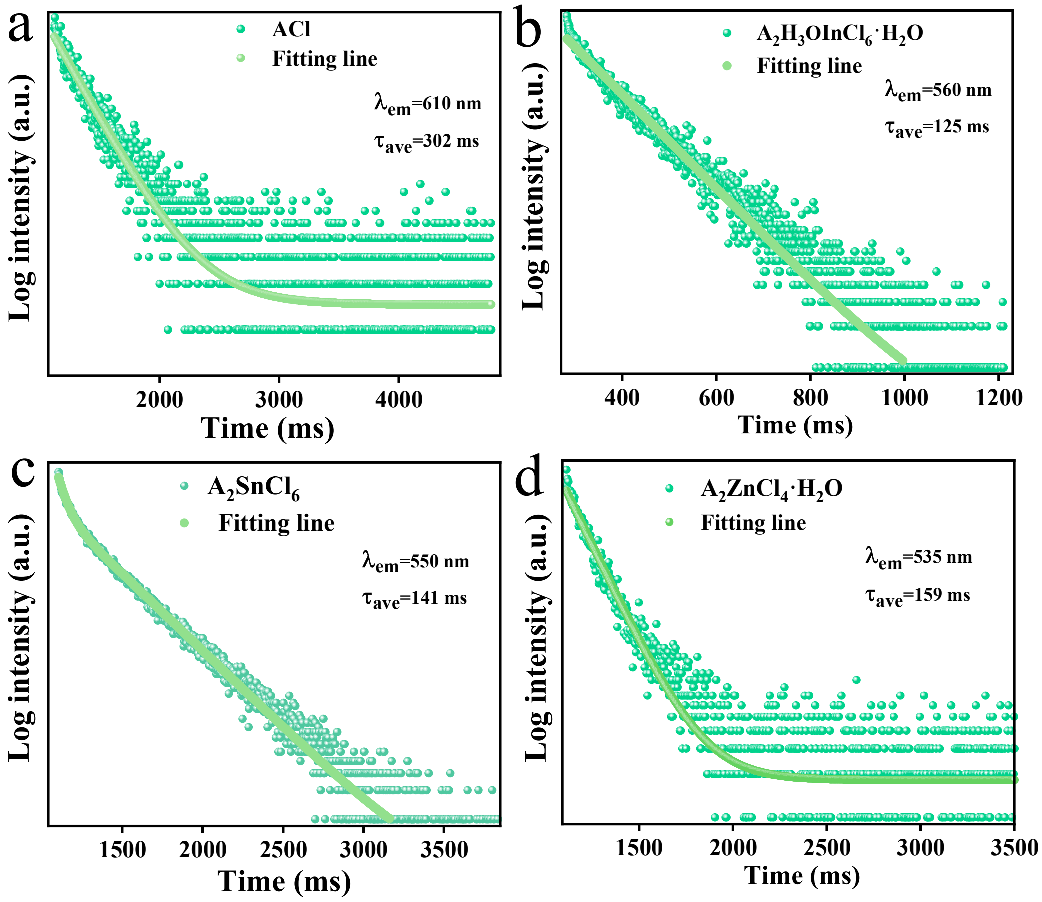

Figure S10. Transient RTP decay curves of (a) ACl, (b) $A_2H_3OInCl_6{\cdot}H_2O$, (c) A_2SnCl_6 and (d) $A_2ZnCl_4{\cdot}H_2O$, recorded at 610, 560, 550 and 535 nm at RT, respectively.

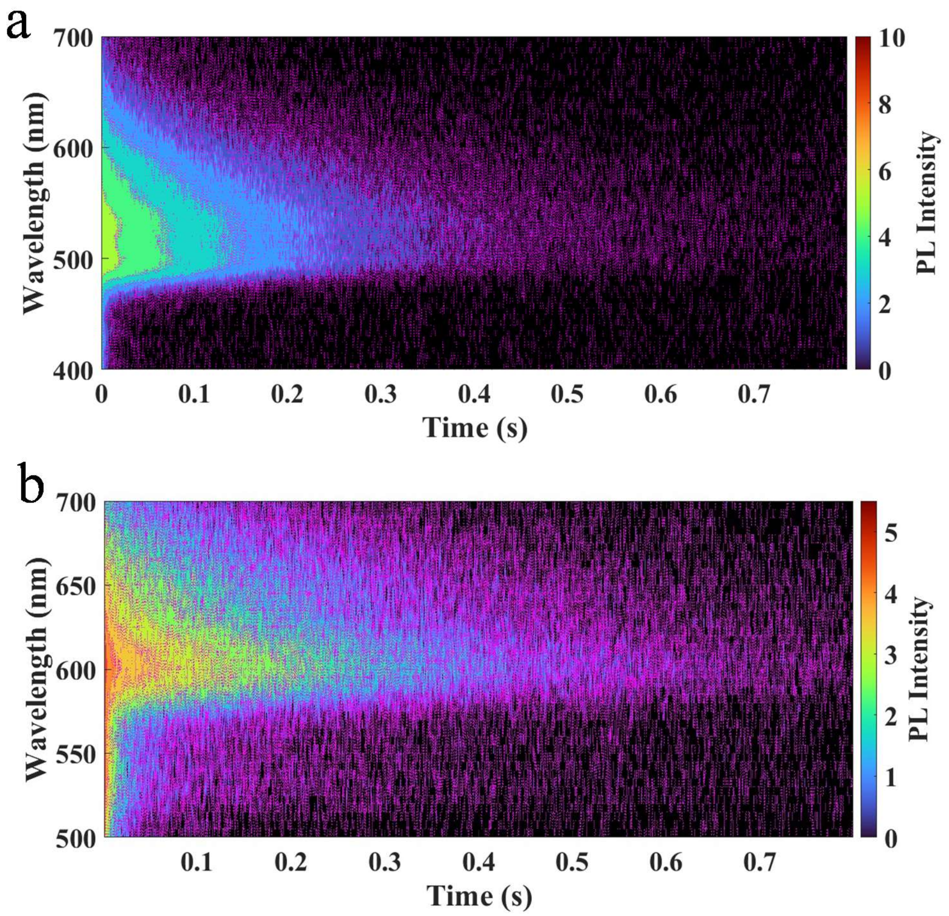

Figure S11. The TRES spectra of (a) $A_2ZnCl_4{\cdot}H_2O$ and (b) ACl, recorded at RT. For the TRES test, a pulsed light source is first used to excite the sample, then the resulting PL attenuation is continuously recorded as a function of the emission wavelength to create a three-dimensional data that includes photoluminescence spectra and time correlation.

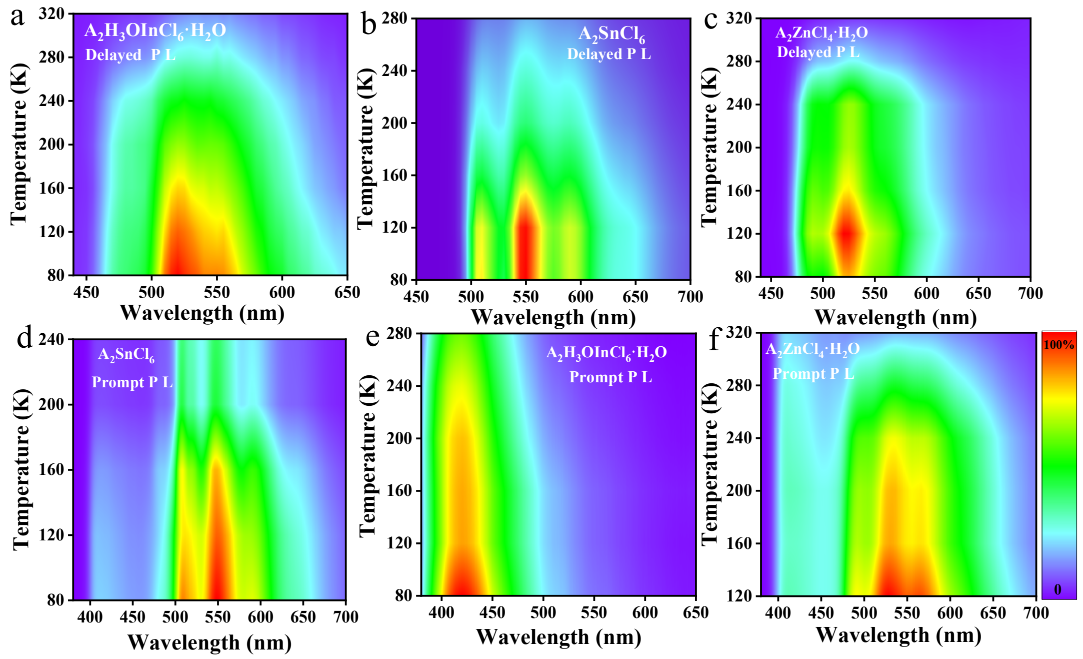

Figure S12. The temperature-dependent delayed PL spectra of (a) $A_2H_3OInCl_6·H_2O$, (b) A_2SnCl_6, (c) $A_2ZnCl_4·H_2O$. The temperature-dependent prompt PL spectra of (d) $A_2H_3OInCl_6·H_2O$, (e) A_2SnCl_6 and (f) $A_2ZnCl_4·H_2O$.

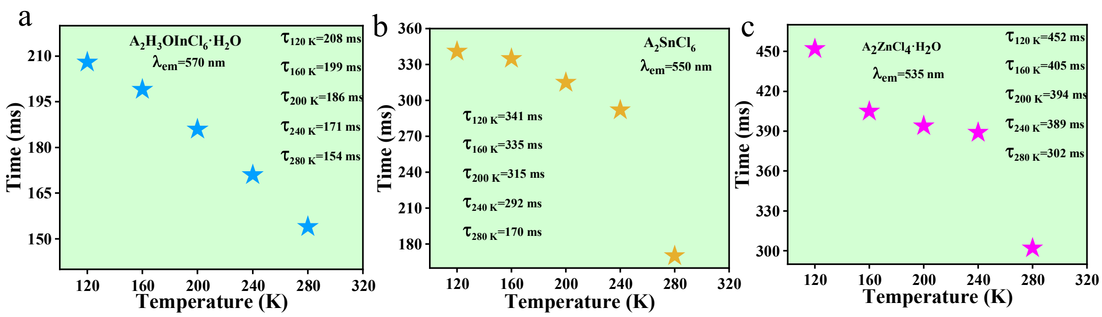

Figure S13. Transient RTP decay curve of (a) $A_2H_3OInCl_6·H_2O$, (b) A_2SnCl_6 and (c) $A_2ZnCl_4·H_2O$, recorded at different temperatures.

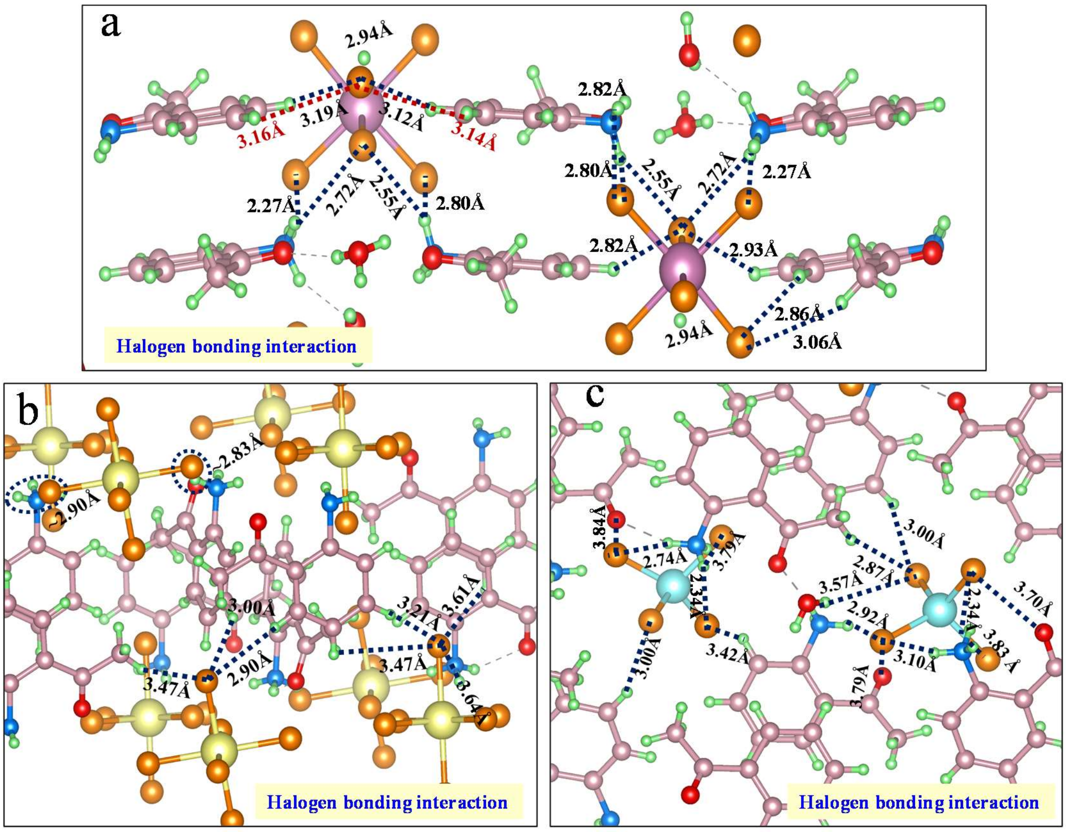

Figure S14. Multiple halogen bonding interactions in (a) $A_2H_3OInCl_6 \cdot H_2O$, (b) A_2SnCl_6 and (c) $A_2ZnCl_4 \cdot H_2O$.

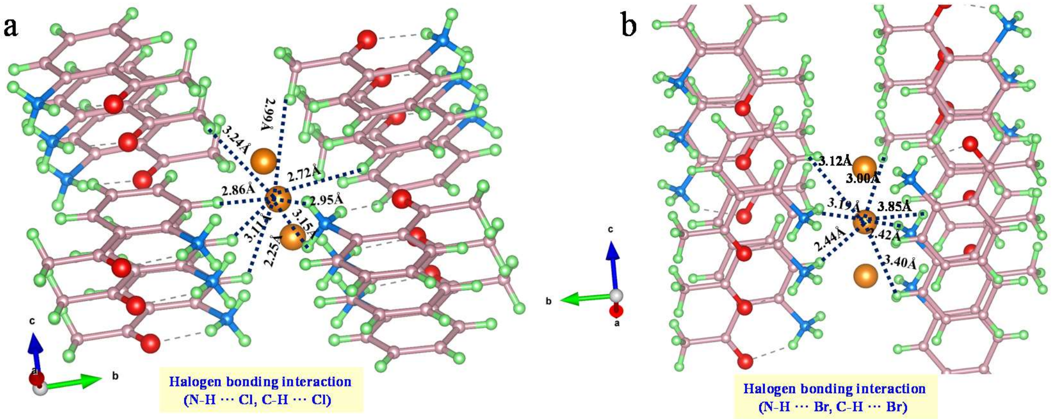

Figure S15. Multiple halogen bonding interactions in (a) ACl and (b) ABr.

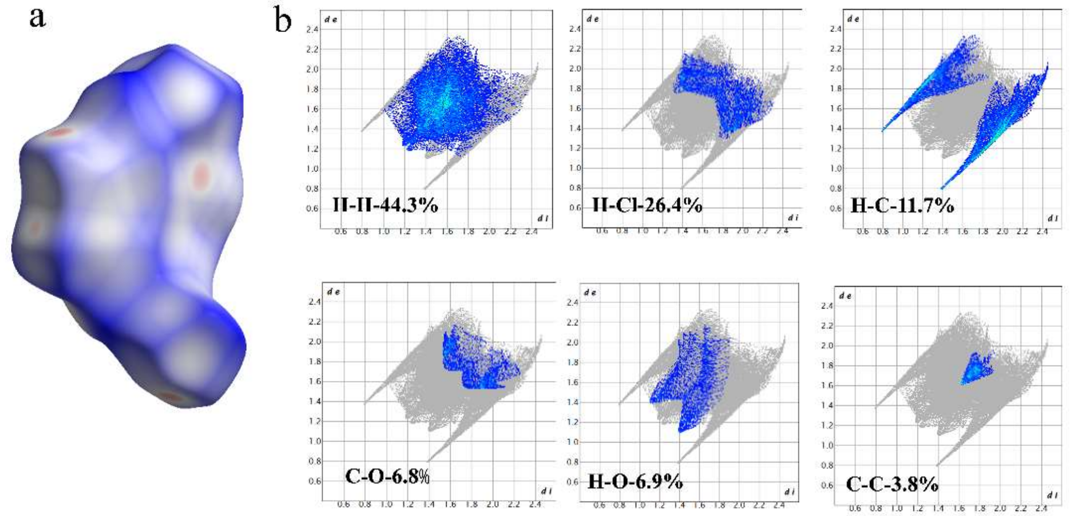

Figure S16. The Hirshfeld surface analyses of ACl. Image showing the Hirshfeld surface of ACl. The Hirshfeld surface is constituted by a series of iso-surfaces consisting of red, white, and blue regions. Red means the intermolecular contact distance is shorter than the van der Waals distance, white means equal and blue means longer. (Two-dimensional fingerprints and relative contributions (in %) to different Hirshfeld surface areas of intermolecular contacts in ACl.)

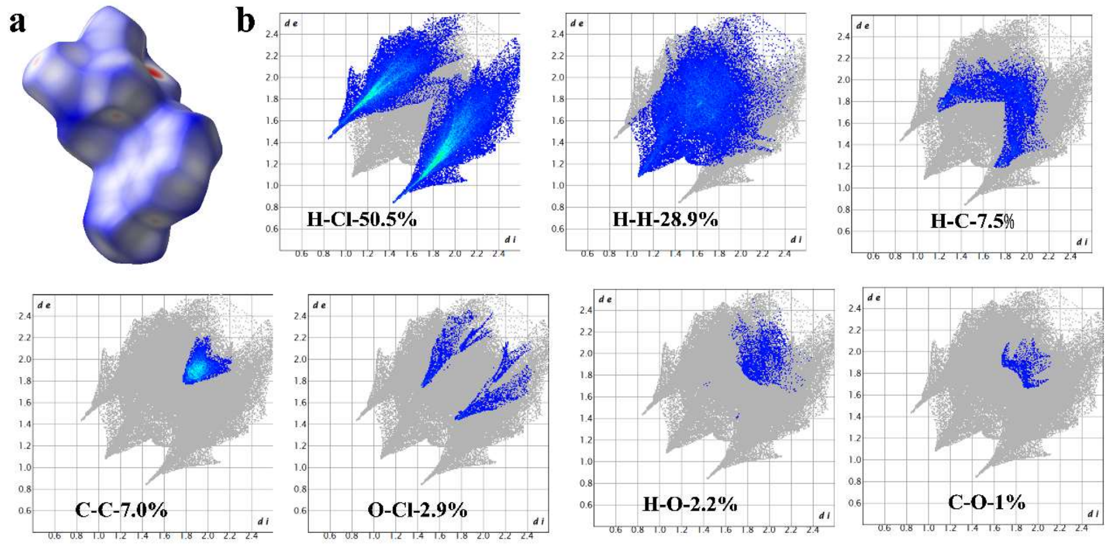

Figure S17. The Hirshfeld surface analyses of $A_2H_3OInCl_6 \cdot H_2O$. Image showing the Hirshfeld surface of $A_2H_3OInCl_6 \cdot H_2O$. (Two-dimensional fingerprints and relative contributions (in %) to different Hirshfeld surface areas of intermolecular contacts in $A_2H_3OInCl_6 \cdot H_2O$.)

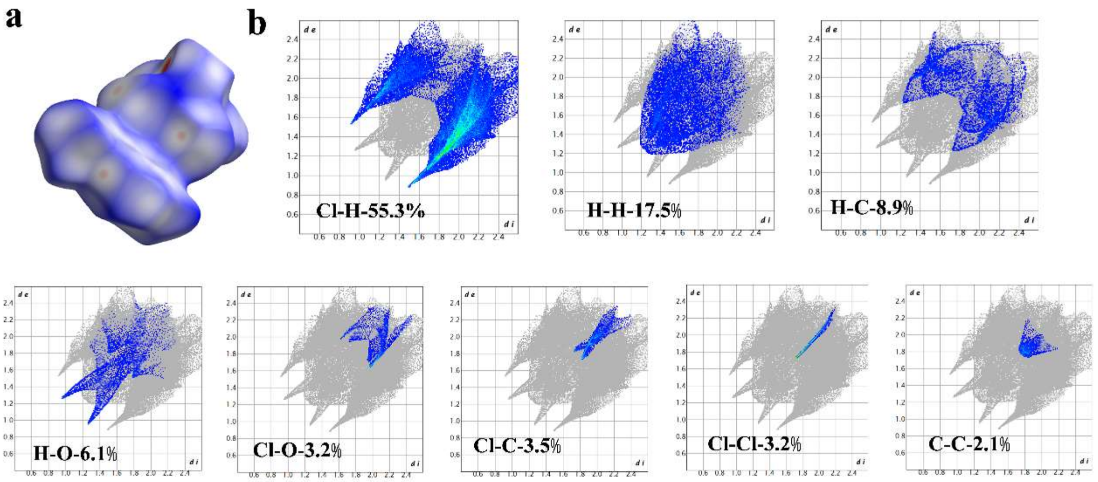

Figure S18. The Hirshfield surface analyses of A_2SnCl_6. Image showing the Hirshfeld surface of A_2SnCl_6. (Two-dimensional fingerprints and relative contributions (in %) to different Hirshfield surface areas of intermolecular contacts in A_2SnCl_6.)

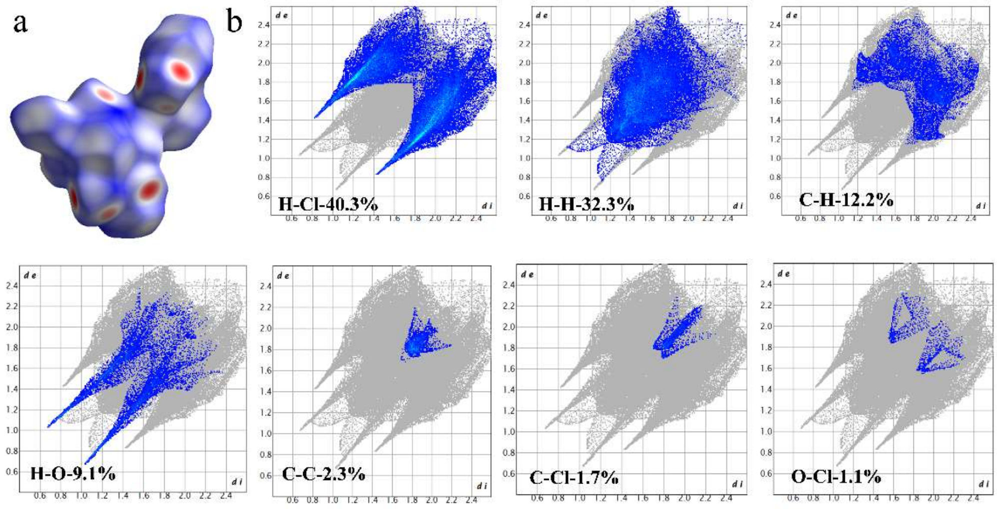

Figure S19. The Hirshfeld surface analyses of $A_2ZnCl_4{\cdot}H_2O$.
Image showing the Hirshfeld surface of $A_2ZnCl_4{\cdot}H_2O$. (Two-dimensional fingerprints and relative contributions (in %) to different Hirshfeld surface areas of intermolecular contacts in $A_2ZnCl_4{\cdot}H_2O$.)

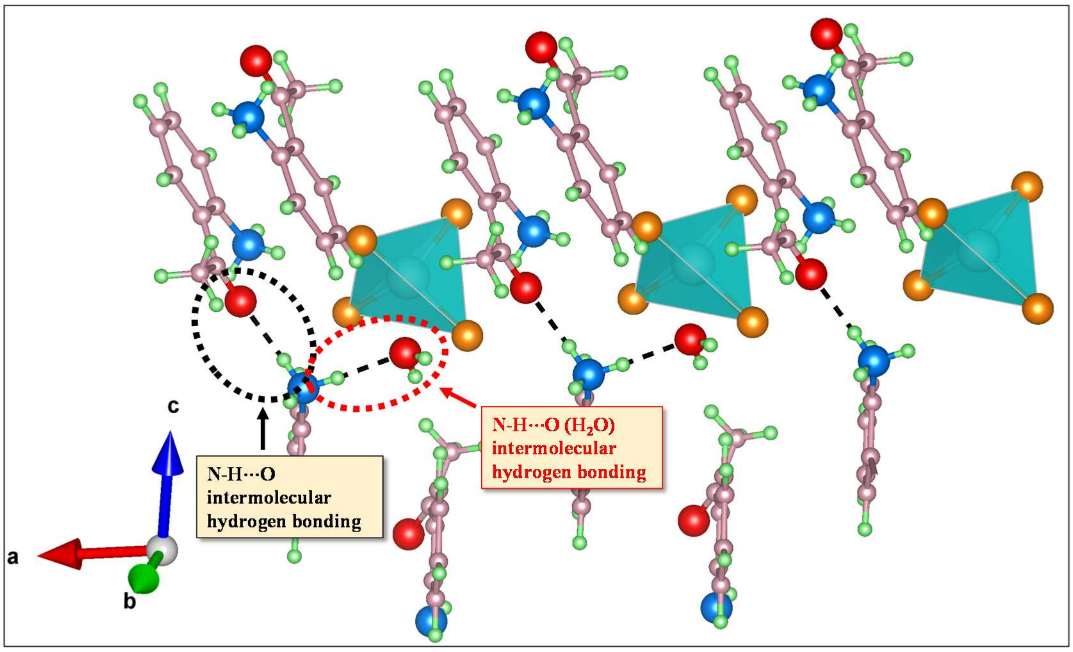

Figure S20. Intermolecular hydrogen bonds in $A_2ZnCl_4 \cdot H_2O$. The red circle: intermolecular hydrogen bonds forming between organic cations and water molecules. The black circle: intermolecular hydrogen bonds forming between adjacent organic cations.

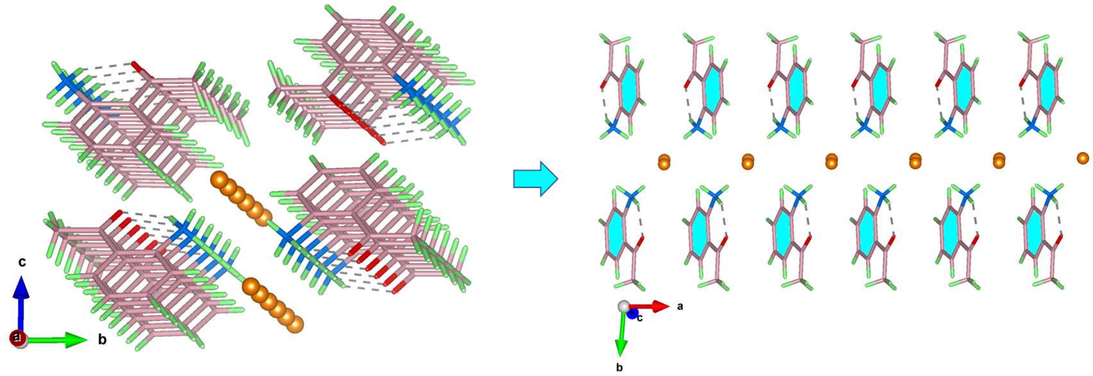

Figure S21. Schematic illustration of the centroid-centroid distances and dihedral angle between two adjacent phenyl rings for (a) ACl and (b) ABr.

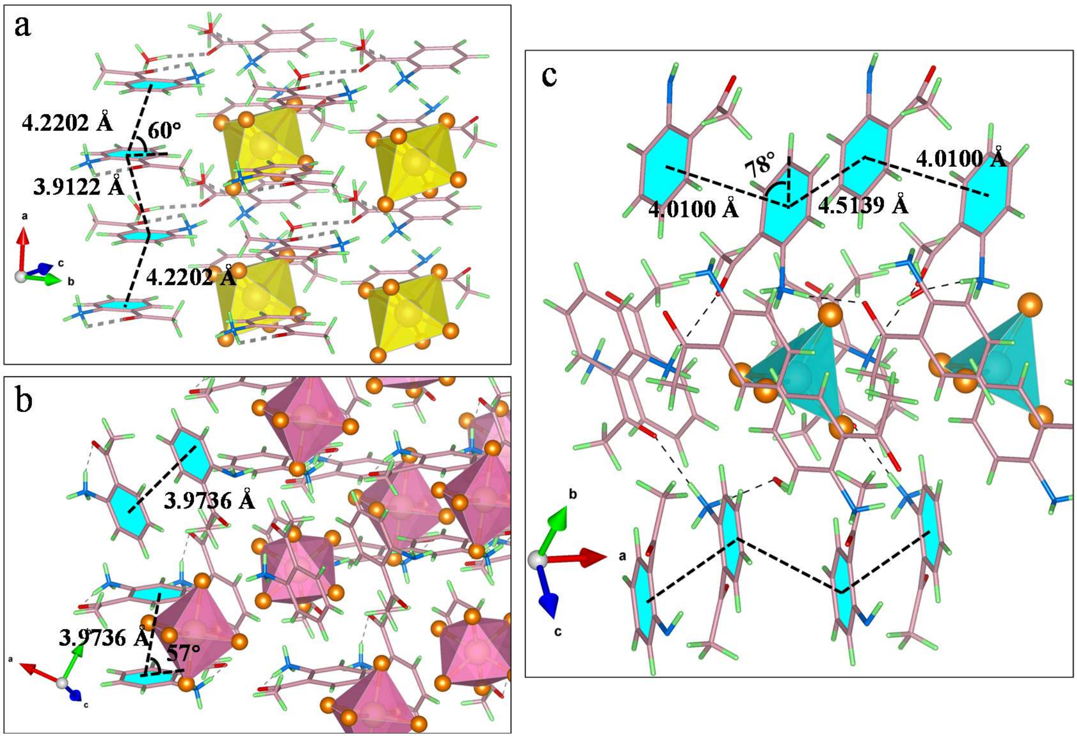

Figure S22. Schematic illustration of the centroid-centroid distances and dihedral angle between two adjacent phenyl rings for (a) $A_2H_3OInCl_6{\cdot}H_2O$, (b) A_2SnCl_6 and (c) $A_2ZnCl_4{\cdot}H_2O$.

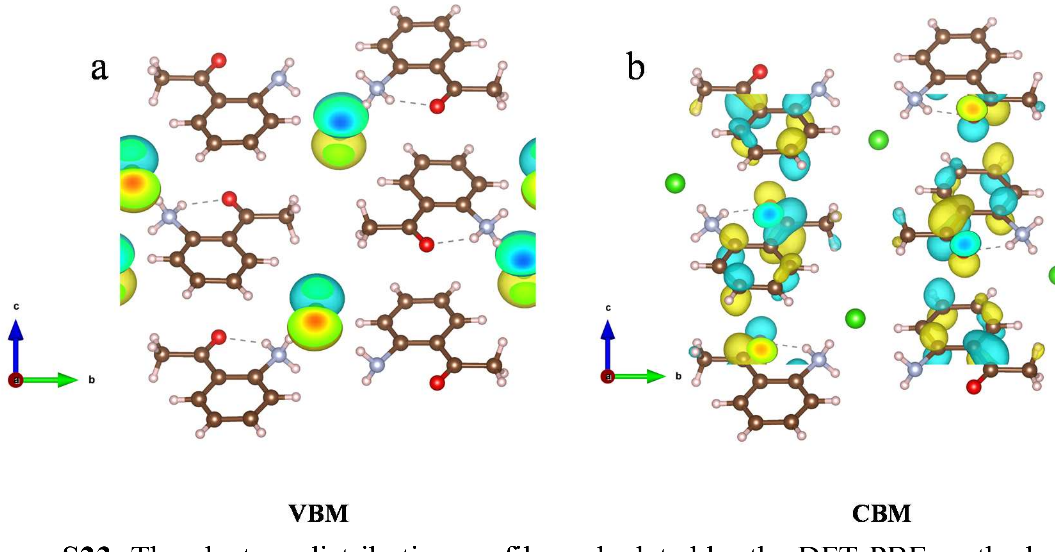

Figure S23. The electron distribution profiles calculated by the DFT-PBE method of (a) VBM and (b) CBM for ACl along *a* axis.

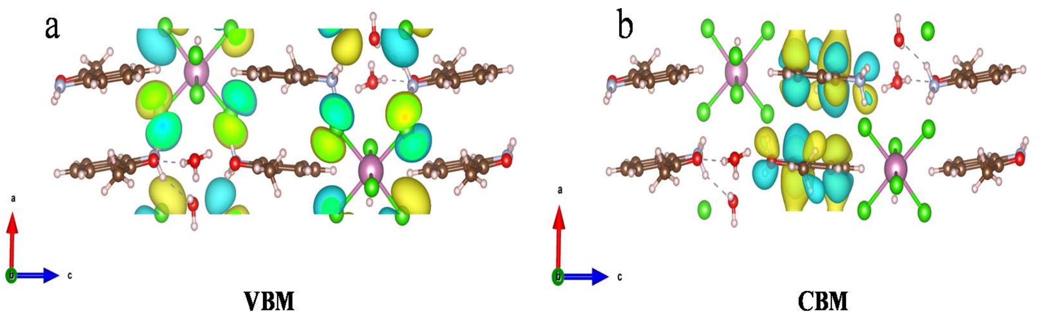

Figure S24. The electron distribution profiles calculated by the DFT-PBE method of (a) VBM and (b) CBM for $A_2H_3OInCl_6 \cdot H_2O$ along *a* axis.

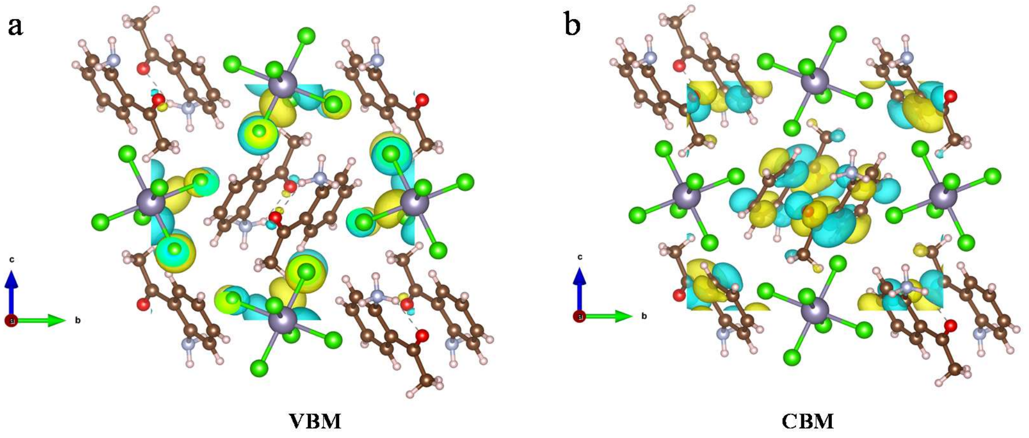

Figure S25. The electron distribution profiles calculated by the DFT-PBE method of (a) VBM and (b) CBM for A_2SnCl_6 along *a* axis.

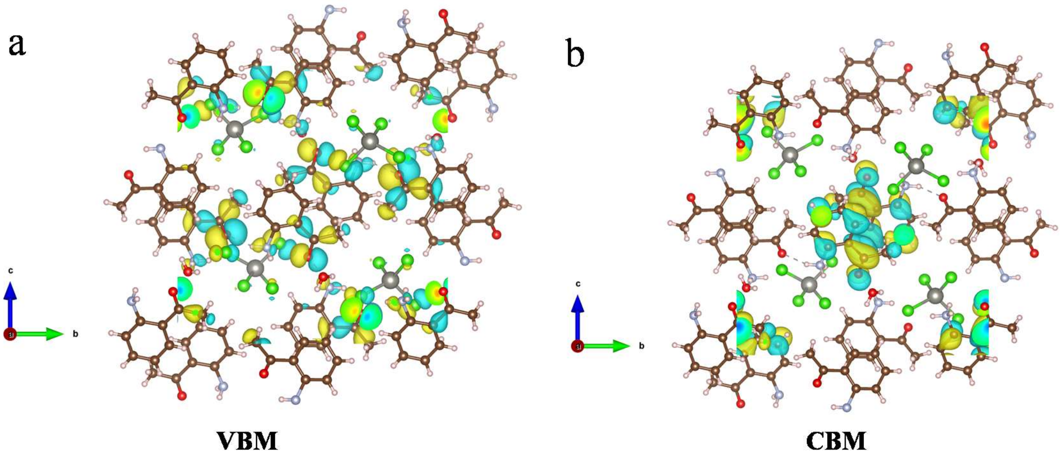

Figure S26. The electron distribution profiles calculated by the DFT-PBE method of (a) VBM and (b) CBM for $A_2ZnCl_4 \cdot H_2O$ along a axis.

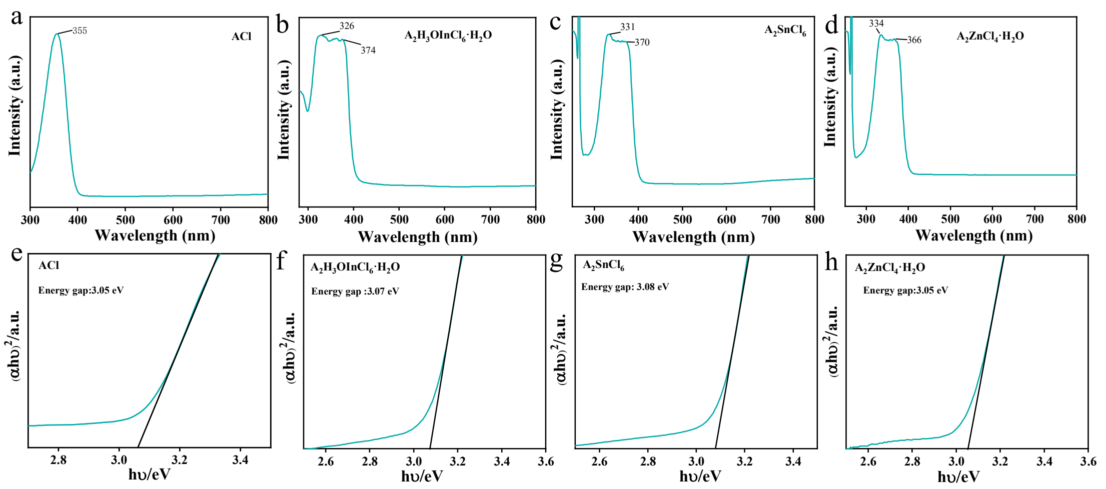

Figure S27. UV-vis absorption spectra of (a) ACl, (b) $A_2H_3OInCl_6 \cdot H_2O$, (c) A_2SnCl_6 and (d) $A_2ZnCl_4 \cdot H_2O$ diluted in dichloromethane solution. The corresponding energy gaps obtained based on Tauc plots of (e) ACl, (f) $A_2H_3OInCl_6 \cdot H_2O$, (g) A_2SnCl_6 and (h) $A_2ZnCl_4 \cdot H_2O$.

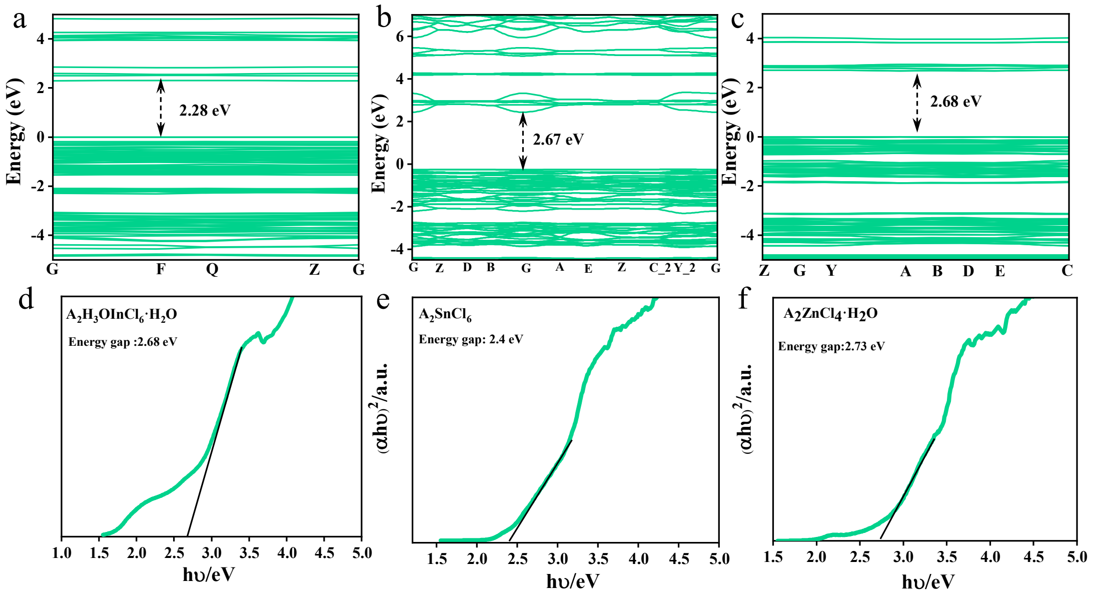

Figure S28. Calculated band structure of (a) $A_2H_3OInCl_6 \cdot H_2O$, (b) A_2SnCl_6 and (c) $A_2ZnCl_4 \cdot H_2O$. Tauc plot curves of (d) $A_2H_3OInCl_6 \cdot H_2O$, (e) A_2SnCl_6, and (f) $A_2ZnCl_4 \cdot H_2O$, calculated based on the experimentally measured UV-vis absorption spectra.

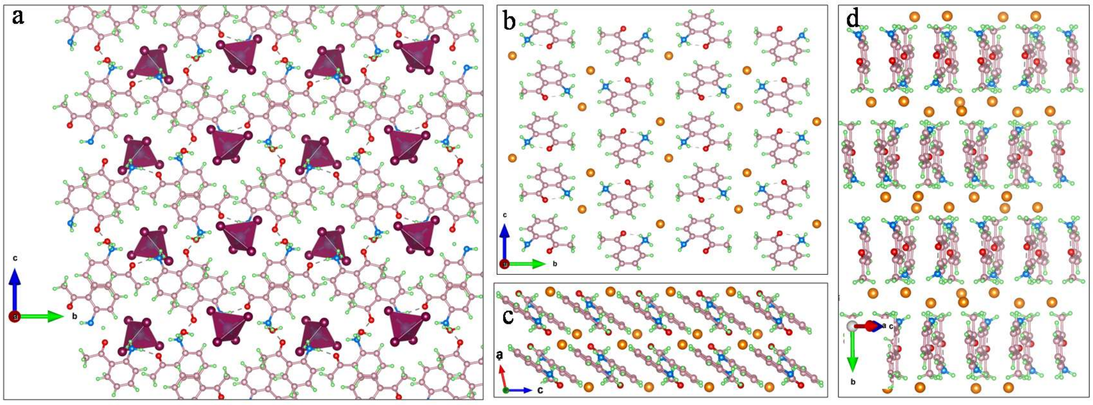

Figure S29. (a) Crystal structure of $A_2ZnBr_4{\cdot}H_2O$ view along the *a* axis. (b-d) Crystal structure of ABr viewed along different directions.

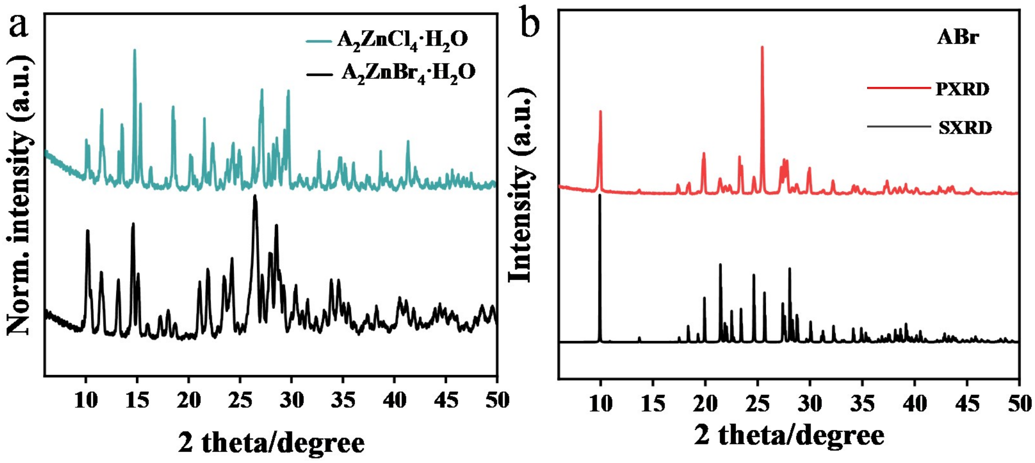

Figure S30. (a) PXRD patterns of $A_2ZnCl_4{\cdot}H_2O$ and $A_2ZnBr_4{\cdot}H_2O$. (b) PXRD and SCXRD patterns of ABr.

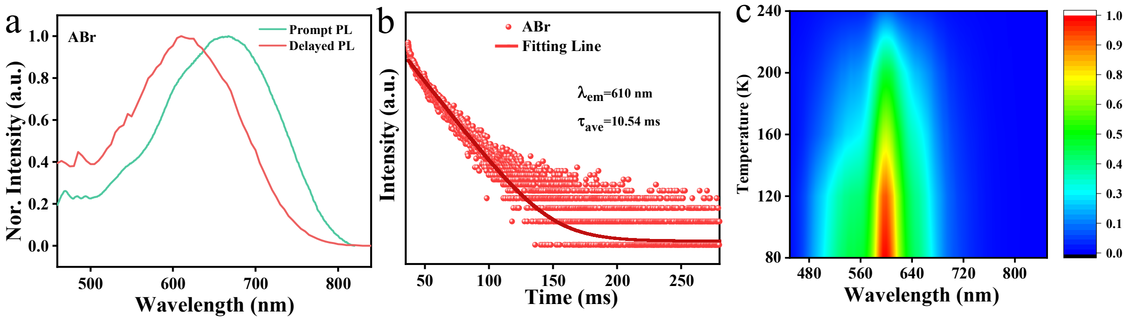

Figure S31. (a) Prompt and delayed spectra of ABr. (b) Transient PL decay curves of ABr, measured at RT. (c) Temperature-dependent delayed spectra of ABr.

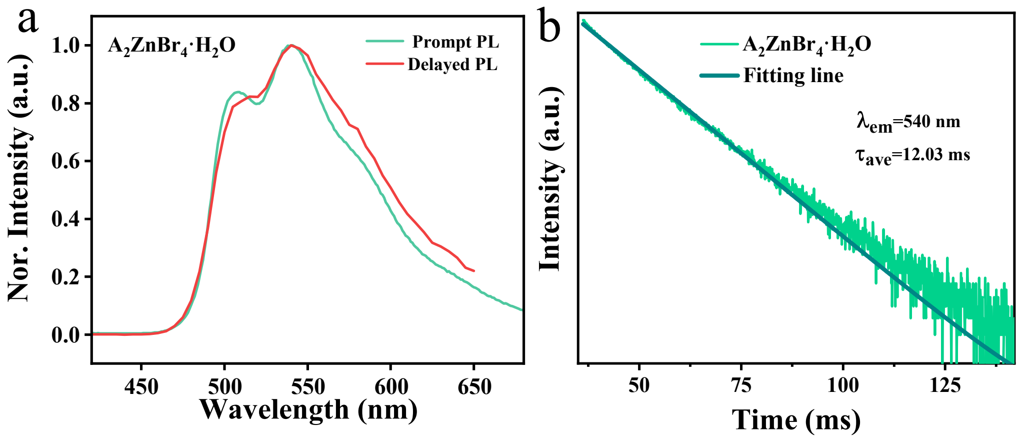

Figure S32. (a) Prompt and delayed spectra of $A_2ZnBr_4·H_2O$. (b) Transient PL decay curves of $A_2ZnBr_4·H_2O$, measured at RT.

Table S1. Single X-ray diffraction data of $A_2H_3OInCl_6\cdot H_2O$, $A_2ZnCl_4\cdot H_2O$, A_2SnCl_6, ACl and ABr.

Compound	$A_2H_3OInCl_6\cdot H_2O$	$A_2ZnCl_4\cdot H_2O$	A_2SnCl_6
Formula weight	636.90	497.52	603.73
Temperature/K	193.00	296.15	296.15
Crystal system	triclinic	monoclinic	monoclinic
Space group	P-1	$P2_1/n$	$P2_1/c$
a/Å	7.3505(6)	7.308(3)	9.3919(6)
b/Å	9.0364(6)	16.775(8)	11.4149(7)
c/Å	18.5560(14)	17.665(8)	11.0818(6)
α/°	89.703(4)	90	90
β/°	88.498(4)	101.133(12)	108.353(2)
γ/°	82.907(4)	90	90
Volume/$Å^3$	1222.67(16)	2124.7(16)	1127.62(12)
Z	2	4	2
$\rho_{calc}g/cm^3$	1.730	1.555	1.778
μ/mm^{-1}	9.430	1.677	1.859
Reflections collected	5088	28478	19440
Goodness-of-fit on F_2	1.035	1.078	1.254
Final R indexes [I>=2σ (I)]	R_1= 0.0694, wR_2= 0.1914	R_1= 0.0781, wR_2= 0.1267	R_1 = 0.0278, wR_2 = 0.0678
Final R indexes	R_1= 0.0844, wR_2= 0.2136	R_1= 0.1386, wR_2= 0.1465	R_1 = 0.0328, wR_2 = 0.0700

Table S2. Single X-ray diffraction data of $A_2ZnBr_4 \cdot H_2O$, A_2SnCl_6, ACl and ABr.

**Compound**	**$A_2ZnBr_4H_2O$**	**ACl**	**ABr**
Formula weight	675.36	171.62	216.08
Temperature/K	193.00	193.00	198.00
Crystal system	monoclinic	monoclinic	monoclinic
Space group	P21/c	P21/c	P21/c
a/Å	7.5012(2)	4.8984(2)	4.9287(2)
b/Å	17.0754(4)	15.8013(6)	16.2486(6)
c/Å	17.8494(6)	10.8025(5)	10.9152(4)
α/°	90	90	90
β/°	101.8150(10)	102.614(2)	102.4850(10)
γ/°	90	90	90
Volume/Å^3	2237.82(11)	815.94(6)	853.47(6)
Z	4	4	4
ρ_{calc}g/cm^3	2.005	1.397	1.682
μ/mm^{-1}	8.257	2.416	6.094
Reflections collected	66056	5613	8238
Goodness-of-fit on F_2	1.051	1.070	1.083
Final R indexes [I>=2σ (I)]	R_1= 0.0209, wR_2= 0.0475	R_1 = 0.0517, wR_2 =0.1422	R_1 = 0.0262, wR_2 = 0.0713
Final R indexes	R_1= 0.0263, wR_2= 0.0492	R_1 = 0.0634, wR_2 =0.1503	R_1 = 0.0275, wR_2 = 0.0719

Table S3. The corresponding CIE coordinate diagrams of $A_2H_3OInCl_6 \cdot H_2O$, A_2SnCl_6, $A_2ZnCl_4 \cdot H_2O$, $A_2ZnBr_4 \cdot H_2O$, ACl and ABr.

Sample	Prompt CIE	Delayed CIE
ACl	(0.3144,0.4606)	(0.6101,0.3866)
ABr	(0.4870,0.4126)	NA
$A_2ZnCl_4 \cdot H_2O$	(0.3553,0.4420)	(0.3167,0.5556)
A_2SnCl_6	(0.4109,0.4619)	(0.4166,0.5524)
$A_2H_3OInCl_6 \cdot H_2O$	(0.1809,0.1522)	(0.4452,0.5060)
$A_2ZnBr_4 \cdot H_2O$	(0.3584,0.5731)	NA

Table S4. Quantum yield of $A_2ZnCl_4 \cdot H_2O$, $A_2ZnBr_4 \cdot H_2O$, $A_3H_3OInCl_6 \cdot HO$, A_2SnCl_6, ABr and ACl, measured at RT.

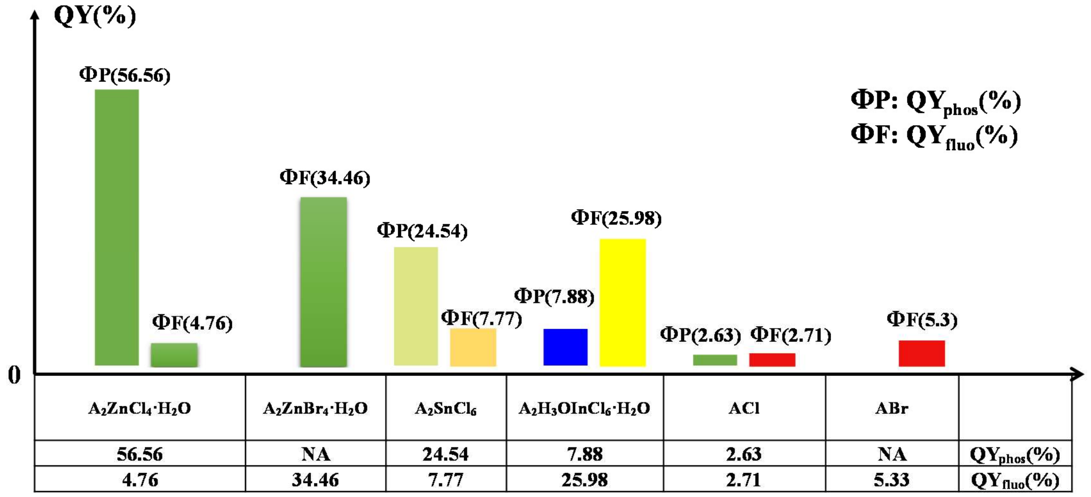

$A_2ZnCl_4 \cdot H_2O$	$A_2ZnBr_4 \cdot H_2O$	A_2SnCl_6	$A_2H_3OInCl_6 \cdot H_2O$	ACl	ABr	
56.56	NA	24.54	7.88	2.63	NA	QY_{phos}(%)
4.76	34.46	7.77	25.98	2.71	5.33	QY_{fluo}(%)

Table S5. Summarized of reported materials with RTP/TADF/LPL emission.

Organic	Compound	λem (nm)	τ	φP (%)	Journal
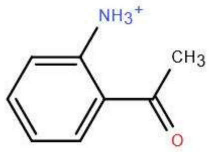	$A_2ZnCl_4{\cdot}H_2O$	535	159ms	57	***This work***
	A_2SnCl_6	575	141ms	25	
	$A_2H_3OInCl_6{\cdot}H_2O$	590	123ms	7.81	
	ACl	610	302ms	2.71	
	ABr	610	10.54ms	NA	
	$A_2ZnBr_4{\cdot}H_2O$	540	12.03ms	NA	
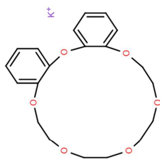	$(KC)_2MnCl_4$	518	2.79ms	7.79	1
	$(KC)_2MnBr_4$	518	0.35ms	38.5	
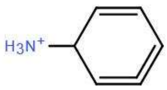	PA_6InCl_9	466	1.18ms	25.2	2
		466	1.22ms	42.8	
	PA_4InCl_7	468	1.19ms	<1	
		467	1.03ms	5.2	
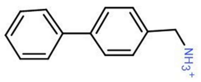	PBA_2ZnBr_4	577	LPL	10.2	3
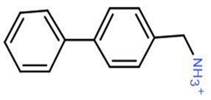	$PBA_3InCl_6{\cdot}H_2O$.	617	290.4ms		4
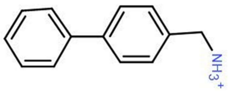	$(BPMA)_2PbBr_4$	527	4.73ms	5.6	5
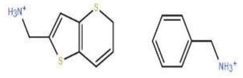	PTPB	560	3.04ms	11.2	6
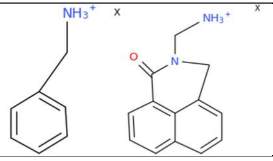	PEPB-NIA5	555	6.46ms	12.6	7
	PEPB-NIA10	555	6.26ms	18.7	
	PEPB-NIA20	555	6.27ms	25.6	
	PEPC-NIA5	550	35.1ms	56.1	
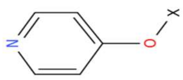	ZnCl2-G	430<br>460<br>480	4.83ms<br>8.46ms<br>13.02ms	11.42	8
	ZnCl2–B	421<br>430<br>450<br>480	96.69ms<br>109.00ms<br>118.09ms<br>120.5ms	24.48	
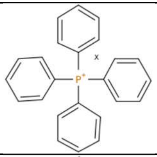	TPP_2ZnCl_4	495	215.5ms	6	9
	TPP_2ZnBr_4	495	37.2ms		
	TPP_2MnCl_4	517	1.97ms	48	
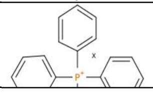	$(Ph_4P)_2Cd_2Cl_6$	500	820ms	23.49	10

	$(Ph_4P)_2Cd_2Br_6$	500	37.85ms	62.79	
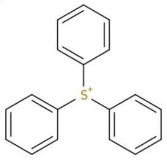	$(Ph_3\ S)_2SnCl_6$	505	297.01ms	3.50	11
	$(Ph_3S)_2Sn_{0.77}Te_{0.33}Cl_6$	520	201ms	1.70	
	$(Ph_3\ S)_2\ ZnCl_4$	535	263.71ms	16.80	
	$(Ph_3S)_2Zn_{0.74}Mn_{0.26}Cl_4$	525	72.61ms	12.70	
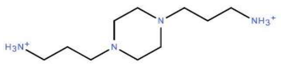	$BAPPZn_2Cl_8$	550	127.73ms		12
	$BAPPZn_2(Cl_{0.9}Br_{0.1})_8$	550	33.32ms		
	$BAPPZn_2(Cl_{0.5}Br_{0.5})_8$	550	9.93ms		
	$BAPPZn_2Br_8$	550	7.84ms		
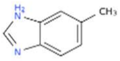	$5\text{-}MBIPbBr_3$	520nm	12ms	26	13
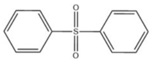	Diphenyl Sulfone	529	43.2ms	2.5	14
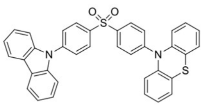	CP-DMF	510	348ms	60.7	
	CP-THF	510	378.9ms	78.8	
	CP-DCM	510	305.6ms	47.5	
	CP-DMSO	510	483ms	62.7	
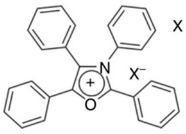	TPO-I	559	48740ns	35	15
	TPO-Br	434	2.52	36.56	
	TPO-Cl	435	1.6ns	20.05	
	TPO-F	420	0.8ns	11.11	
	TPO-P	422	1.02ns	18.58	
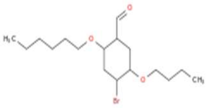 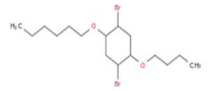	Br6A/Br6	510	8.3ms	55	16
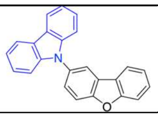	CZ-DBF	430	650ms	14.3	17
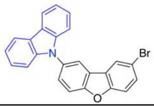	CZ-DBFBr	430	540ms	41.2	
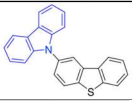	CZ-DBT	430	450ms	10.1	
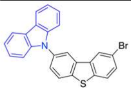	CZ-DBTBr	430	420ms	12.1	
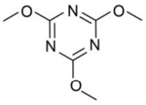	TMOT	465	59.88ms	31.2	18
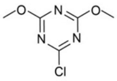	DMOT	430	2450ms	7.2	
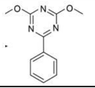	MOPT	521	481ms	0.2	
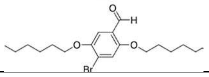	Br6A	5.4 ms	2.9ms		19
	α-AlF3	450	900ms	4.22	20

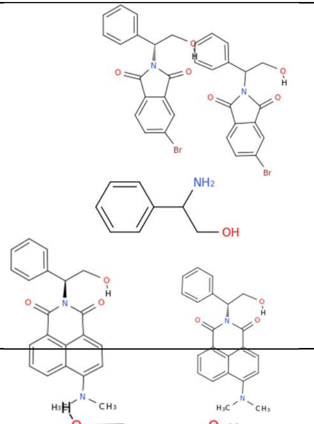	RS-4FBrBI	488	6.50ms	<0.1	21
	RS-PgBrBI	493	7.24ms	<0.1	
	RS-4FMNNI	510	9.68ns	75.7	
	RS-PgMNNI	518	9.69ns	68.1	
	R@R	615	73.31ms		
	S@R	618	44.79ms		
	S@S	617	70.55ms		
	R@S	618	41.9ms		
	R-4FBrBI	482	0.62ms		
	S-4FBrBI	482	0.85ms		
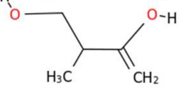	(S, S)-DAACH	400-700	587ms	2.54	22
	(R, R)-DAACH	350-500	2.3ms	3.38	
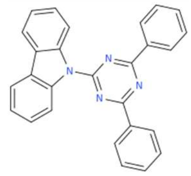	DPhCzT	416<br>440<br>465<br>493<br>530<br>573	374 ms<br>573ms<br>676ms<br>746ms<br>1066ms<br>1052ms	1.25	23
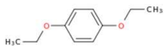	DEOPh	515<br>547	644ms<br>643ms	0.3	
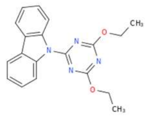	DECzT	529<br>574	1281ms<br>1347ms	0.6	
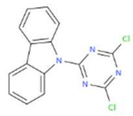	CzDCIT	543<br>591	431ms<br>453ms	2.1	
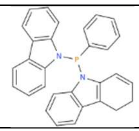	DCZPhP	587<br>644	208.6ms<br>207.1ms	0.08	
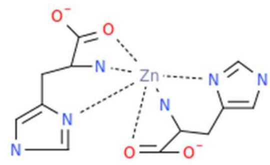	Zn-L-1 SG	430 ~ 500	356.7ms	18.32	24
	Zn-L-2 SG	460 ~ 545	307.9ms	15.21	
	Zn-L-3 SG	430 ~500	224.2ms	11.77	
	Zn-L-4 SG	465 ~ 555	207.6ms	9.54	
	Zn-D-2 SG	529 ~ 579	315.5ms	16.02	
	Zn-L-RB-1 SG	400–537 615	176.3ms	20.31	
	Zn-L-RB-2 SG	416–537 678	141.9ms	21.59	
	Zn-L crystal	529 ~579	6.1ms	6.87	
	Zn-L-1 SG	430 ~ 500	356.7ms	18.32	

Table S6. The K_{ISC} of $A_2ZnCl_4{\cdot}H_2O$, A_2SnCl_6, $A_2H_3OInCl_6{\cdot}H_2O$ and ACl.

Sample	$QY_{phos.}$ (%)	τ_f (ns)	K_{isc} (s^{-1})
$A_2ZnCl_4{\cdot}H_2O$	56.56	1.66	3.4×10^{8}
A_2SnCl_6	24.54	5.4	4.5×10^{7}

$A_2H_3OInCl_6 \cdot H_2O$	7.88	6.88	1.1×10^{7}
ACl	2.63	11.67	2.3×10^{6}

K_{isc} = $QY_{Phos.}/\tau_f$; $QY_{Phos.}$ = absolute quantum yield of phosphorescence; τ_f = lifetime of fluorescence;

Table S7-1. Parameters of the π–π interactions in ACl.

Cg(I) Res(I) > Cg(J)	Cg-Cg (Å)	Alpha (deg)	Beta (deg)	Gamma (deg)	CgIPerp (Å)	CgJPerp (Å)	Slippage (Å)
Cg1 [1] -> Cg1	4.891(12)	0.02(10)	48.9	48.9	3.217(8)	3.217(8)	3.694
Cg1 [1] -> Cg1	4.898(12)	0.02(10)	48.9	48.9	3.211(8)	3.211(8)	3.694
Cg1 [1] -> Cg1	5.462(12)	11.25(10)	53.5	55.6	3.083(8)	3.246(8)	4.393
Cg1 [1] -> Cg1	5.462(12)	11.25(10)	55.6	53.5	3.246(8)	3.086(8)	4.507

Table S7-2. Parameters of the π - π interactions in $A_2H_3OInCl_6 \cdot H_2O$.

Cg(I) Res(I) > Cg(J)	Cg-Cg (Å)	Alpha (deg)	Beta (deg)	Gamma (deg)	CgIPerp (Å)	CgJPerp (Å)	Slippage (Å)
Cg1 [1] -> Cg1	3.951(7)	0.0(6)	21.1	21.1	3.687(5)	3.687(5)	1.419
Cg1 [1] -> Cg1	4.095(7)	0.0(6)	29	29	3.582(5)	3.582(5)	1.984
Cg2 [2] -> Cg2	3.936(7)	0.0(6)	20.8	20.8	3.681(5)	3.680(5)	1.395
Cg2 [2] -> Cg2	4.234(7)	0.0(6)	31.1	31.1	3.627(5)	3.628(5)	2.185

Table S7-3. Parameters of the π - π interactions in $A_2ZnCl_4 \cdot H_2O$.

Cg(I) Res(I) > Cg(J)	Cg-Cg (Å)	Alpha (deg)	Beta (deg)	Gamma (deg)	CgIPerp (Å)	CgJPerp (Å)	Slippage (Å)
Cg1 [1] -> Cg1	4.975(4)	0.0(3)	50.9	50.9	3.140(2)	3.140(2)	3.859
Cg1 [1] -> Cg1	5.466(4)	0.0(3)	49.1	49.1	3.581(2)	3.581(2)	4.13
Cg2 [2] -> Cg2	3.988(4)	0.0(3)	29.1	29.1	3.485(2)	3.485(2)	1.939

Cg2 [2] -> Cg2	4.496(4)	0.0(3)	38.9	38.9	3.498(2)	3.497(2)	2.825

Table S 7-4. Parameters of the π - π interactions in A_2SnCl_6.

Cg(I) Res(I) > Cg(J)	Cg-Cg (Å)	Alpha (deg)	Beta (deg)	Gamma (deg)	CgIPerp, (Å)	CgJPerp (Å)	Slippage (Å)
Cg1 [1] -> Cg1	3.992(2)	0.0(2)	29.2	29.2	3.4863(17)	3.4862(17)	1.945
Cg1 [1] -> Cg1	5.881(2)	79.7(2)	32.8	71	1.9190(17)	4.9451(17)	

Table S7-5. Parameters of the π - π interactions in ABr.

Cg(I) Res(I) > Cg(J)	Cg-Cg (Å)	Alpha (deg)	Beta (deg)	Gamma (deg)	CgIPerp (Å)	CgJPerp (Å)	Slippage (Å)
Cg1 [1] -> Cg1	4.9287(11)	0.03(9)	48.7	48.7	3.2500(8)	3.2500(7)	3.705
Cg1 [1] -> Cg1	4.9287(11)	0.03(9)	48.7	48.7	3.2501(7)	3.2501(8)	3.705
Cg1 [1] -> Cg1	5.5162(11)	9.86(9)	55.4	53.6	3.2706(8)	3.1327(8)	4.54
Cg1 [1] -> Cg1	5.5164(11)	9.86(9)	53.6	55.4	3.1327(7)	3.2707(7)	4.442

Table S7-6. Parameters of the π - π interactions in $A_2ZnBr_4 \cdot H_2O$.

Cg(I) Res(I) > Cg(J)	Cg-Cg (Å)	Alpha (deg)	Beta (deg)	Gamma (deg)	CgIPerp (Å)	CgJPerp (Å)	Slippage (Å)
Cg1 [1] -> Cg1	5.1228(12)	0.00(11)	53.8	53.8	3.0288(9)	3.0289(9)	4.131
Cg1 [1] -> Cg1	5.7866(13)	0.00(11)	49.3	49.3	3.7717(9)	3.7717(9)	4.389
Cg2 [2] -> Cg2	3.9477(13)	0.00(11)	28.7	28.7	3.4619(9)	3.4618(9)	1.897
Cg2 [2] -> Cg2	4.7268(13)	0.00(11)	40.3	40.3	3.6072(9)	3.6072(9)	3.055

Cg(I) = Plane number I (= ring number in () above)
Alpha = Dihedral Angle between Planes I and J (Deg)
Beta = Angle Cg(I)-->Cg(J) or Cg(I)-->Me vector and normal to plane I (Deg)
Gamma= Angle Cg(I)-->Cg(J) vector and normal to plane J (Deg)
Cg-Cg = Distance between ring Centroids (Ang.)
CgI_Perp = Perpendicular distance of Cg(I) on ring J (Ang.)
CgJ_Perp = Perpendicular distance of Cg(J) on ring I (Ang.)
Slippage = Distance between Cg(I) and Perpendicular Projection of Cg(J) on Ring I (Ang)

Table S8-1. Hydrogen-bond geometry in $A_2H_3OInCl_6{\cdot}H_2O$.

Donor ··· H···Acceptor	d-D - H (Å)	d-H···A (Å)	d-D···A (Å)	∠D - H···A (deg)
1 N1···H1B···O4	0.91	2.01	2.834(14)	150
Intra 1 N1···H1C···O1	0.91	2.14	2.631(15)	113
Intra 2 N2···H2A···O2	0.91	2.07	2.639(15)	119
2 N2···H2A···O4	0.91	2.17	2.883(13)	135
4 O3···H3A···O2	0.87	2.14	2.882(13)	144
4 O3···H3B···O1	0.87	2.00	2.854(13)	168

Table S8-2. Hydrogen-bond geometry in A_2SnCl_6.

Donor ··H···Acceptor	d-D - H (Å)	d-H···A (Å)	d-D···A (Å)	∠D - H···A (deg)
Intra 1 N006···H00B···O005	0.89	1.95	2.643(5)	134
1 N006···H00B···O005	0.89	2.29	2.915(4)	127'

Table S8-3. Hydrogen-bond geometry in $A_2ZnCl_4{\cdot}H_2O$.

Donor ··· H···Acceptor	d-D - H (Å)	d-H···A (Å)	d-D···A (Å)	∠D - H···A (deg)
Intra 2N008···00H···O007	0.89	2.17	2.692(5)	117'
2N008···00J···O00B	0.89	1.83	2.719(7)	176

2N008···H00M···O006	0.89	1.97	2.858(6)	173
1N009···H00A···O007	0.89	2.09	2.868(5)	146
Intra 1N009···H00B···O006	0.89	2.34	2.645(5)	100'

Table S8-4. Hydrogen-bond geometry in ACl.

**Donor ···H···Acceptor**	**d-D - H (Å)**	**d-H···A (Å)**	**d-D···A (Å)**	**∠D - H···A (deg)**
Intra 1N1···H1C···O1	0.91	1.91	2.677(2)	141
1C3···H3···O1	0.95	2.59	3.243(3)	126

Table S8-5. Hydrogen-bond geometry in $A_2ZnBr_4 \cdot H_2O$.

**Donor ···H···Acceptor**	**d-D - H (Å)**	**d-H···A (Å)**	**d-D···A (Å)**	**∠D - H···A (deg)**
Intra 1N1···H1B···O1	0.91	2.28	2.669(2)	106'
1 N1···H1C···O2	0.91	2.00	2.862(2)	158
2 N2···H2A···O1	0.91	1.97	2.873(2)	173
2 N2···H2B···O3	0.91	1.82	2.729(3)	172
Intra 2N2···H2C···O2	0.91	2.17	2.691(2)	116

Table S8-6. Hydrogen-bond geometry in ABr.

**Donor ···H···Acceptor**	**d-D - H (Å)**	**d-H···A (Å)**	**d-D···A (Å)**	**∠D - H···A (deg)**
Intra 1N1···H1B···O1	0.91	1.90	2.668(2)	141

Supplementary References:

1. J. Zhao, T. Zhang, X. Y. Dong, M. E. Sun, C. Zhang, X. Li, Y. S. Zhao and S. Q. Zang, *Journal of the American Chemical Society*, 2019, **141**, 15755-15760.
2. S. Feng, Y. Ma, S. Wang, S. Gao, Q. Huang, H. Zhen, D. Yan, Q. Ling and Z. Lin, *Angewandte Chemie International Edition*, 2022, **61**, e202116511.
3. H. Gong, H. Yu, Y. Zhang, L. Feng, Y. Tian, G. Cui and H. Fu, *Angewandte Chemie International Edition*, 2023, **62**, e202219085.
4. H. Yu, H. Gong, Z. Hua, Y. Zhang, W. Sun, S. Gong, G. Cui, Y. Tian and H. Fu, *Science China Chemistry*, 2023, **66**, 2576-2582.
5. H. Hu, D. Zhao, Y. Gao, X. Qiao, T. Salim, B. Chen, E. E. M. Chia, A. C. Grimsdale and Y. M. Lam, *Chemistry of Materials*, 2019, **31**, 2597-2602.
6. H. Hu, F. Meier, D. Zhao, Y. Abe, Y. Gao, B. Chen, T. Salim, E. E. M. Chia, X. Qiao, C. Deibel and Y. M. Lam, *Advanced Materials*, 2018, **30**, e1707621.
7. S. Yang, D. Wu, W. Gong, Q. Huang, H. Zhen, Q. Ling and Z. Lin, *Chemical Science*, 2018, **9**, 8975-8981.
8. B. Zhou, G. Xiao and D. Yan, *Advanced Materials*, 2021, **33**, e2007571.
9. L. J. Xu, A. Plaviak, X. S. Lin, M. Worku, Q. Q. He, M. Chaaban, B. J. Kim and B. W. Ma, *Angewandte Chemie International Edition*, 2020, **59**, 23067-23071.
10. S. Liu, X. Fang, B. Lu and D. Yan, *Nature Communications*, 2020, **11**, 4649.
11. Z. Luo, Y. Liu, Y. Liu, C. Li, Y. Li, Q. Li, Y. Wei, L. Zhang, B. Xu, X. Chang and Z. Quan, *Advanced Materials*, 2022, **34**, e2200607.
12. J. H. Wei, W. T. Ou, J. B. Luo and D. B. Kuang, *Angewandte Chemie International Edition*, 2022, **61**, e202207985.
13. Y. Han, Y. W. Dong, H. Gu, T. Cheng, Y. P. Xie, Y. F. Lin, G. C. Xing, J. Yin and B. B. Cui, *Small Structures*, 2022, **3**, 2200110.
14. W. Li, Q. Huang, Z. Mao, X. He, D. Ma, J. Zhao, J. W. Y. Lam, Y. Zhang, B. Z. Tang and Z. Chi, *Nature Communications*, 2022, **13**, 7423.
15. J. Wang, X. Gu, H. Ma, Q. Peng, X. Huang, X. Zheng, S. H. P. Sung, G. Shan, J. W. Y. Lam, Z. Shuai and B. Z. Tang, *Nature Communications*, 2018, **9**, 2963.
16. O. Bolton, K. Lee, H.-J. Kim, K. Y. Lin and J. Kim, *Nature Chemistry*, 2011, **3**, 205-210.
17. W. Zhao, T. S. Cheung, N. Jiang, W. Huang, J. W. Y. Lam, X. Zhang, Z. He and B. Z. Tang, *Nature Communications*, 2019, **10**, 1595.
18. L. Gu, H. Shi, L. Bian, M. Gu, K. Ling, X. Wang, H. Ma, S. Cai, W. Ning, L. Fu, H. Wang, S. Wang, Y. Gao, W. Yao, F. Huo, Y. Tao, Z. An, X. Liu and W. Huang, *Nature Photonics*, 2019, **13**, 406-411.
19. Kenry, C. Chen and B. Liu, *Nature Communications*, 2019, **10**, 2111.
20. P. Cao, H. Zheng and P. Wu, *Nature Communications*, 2022, **13**, 5712.
21. B. Chen, W. Huang and G. Zhang, *Nature Communications*, 2023, **14**, 1514.
22. H. Li, J. Gu, Z. Wang, J. Wang, F. He, P. Li, Y. Tao, H. Li, G. Xie, W. Huang, C. Zheng and R. Chen, *Nature Communications*, 2022, **13**, 429.
23. Z. An, C. Zheng, Y. Tao, R. Chen, H. Shi, T. Chen, Z. Wang, H. Li, R. Deng, X. Liu and W. Huang, *Nature Materials*, 2015, **14**, 685-690.
24. F. Nie, K. Z. Wang and D. Yan, *Nature Communications*, 2023, **14**, 1654.
